# Pre-Therapeutic Sarcopenia among Cancer Patients: An Up-to-Date Meta-Analysis of Prevalence and Predictive Value during Cancer Treatment

**DOI:** 10.3390/nu15051193

**Published:** 2023-02-27

**Authors:** Anne-Laure Couderc, Evelyne Liuu, Pascaline Boudou-Rouquette, Johanne Poisson, Maxime Frelaut, Coline Montégut, Soraya Mebarki, Romain Geiss, Zoé ap Thomas, Aurélien Noret, Monica Pierro, Capucine Baldini, Elena Paillaud, Frédéric Pamoukdjian

**Affiliations:** 1 Internal Medicine Geriatrics and Therapeutic Unit, APHM, 13009 Marseille, France; 2 CNRS, EFS, ADES, Aix-Marseille University, 13015 Marseille, France; 3 Department of Geriatrics, CHU Poitiers, 86000 Poitiers, France; 4 CIC1402 INSERM Unit, Poitiers University Hospital, 86000 Poitiers, France; 5 Ariane Program, Department of Medical Oncology, Cochin Hospital, Paris Cancer Institute CARPEM, APHP, 75014 Paris, France; 6 INSERM U1016-CNRS UMR8104, Cochin Institute, Paris Cancer Institute CARPEM, Paris Cité University, 75015 Paris, France; 7 Department of Geriatrics, Georges Pompidou European Hospital, Paris Cancer Institute CARPEM, APHP, 75015 Paris, France; 8 Faculty of Health, Paris Cité University, 75006 Paris, France; 9 Department of Medical Oncology, Gustave Roussy Institute, 94805 Villejuif, France; 10 Coordination Unit for Geriatric Oncology (UCOG), PACA West, 13009 Marseille, France; 11 Department of Medical Oncology, Curie Institute, 92210 Saint-Cloud, France; 12 Department of Cancer Medicine, Gustave Roussy Institute, 94805 Villejuif, France; 13 Drug Development Department, Gustave Roussy Institute, 94805 Villejuif, France; 14 INSERM, IMRB, Clinical, Epidemiology and Ageing, Université Paris-Est Creteil, 94010 Creteil, France; 15 Department of Geriatrics, Avicenne Hospital, APHP, 93000 Bobigny, France; 16 INSERM UMR_S942 Cardiovascular Markers in Stressed Conditions MASCOT, Sorbonne Paris Nord University, 93000 Bobigny, France

**Keywords:** sarcopenia, cancer, prevalence, risk factor, survival, post-operative complications, toxicities, hospital-acquired infections, disability

## Abstract

This study will address the prevalence of pre-therapeutic sarcopenia (PS) and its clinical impact during cancer treatment among adult cancer patients ≥ 18 years of age. A meta-analysis (MA) with random-effect models was performed via a MEDLINE systematic review, according to the PRISMA statement, focusing on articles published before February 2022 that reported observational studies and clinical trials on the prevalence of PS and the following outcomes: overall survival (OS), progression-free survival (PFS), post-operative complications (POC), toxicities (TOX), and nosocomial infections (NI). A total of 65,936 patients (mean age: 45.7–85 y) with various cancer sites and extensions and various treatment modes were included. Mainly defined by CT scan-based loss of muscle mass only, the pooled prevalence of PS was 38.0%. The pooled relative risks were 1.97, 1.76, 2.70, 1.47, and 1.76 for OS, PFS, POC, TOX, and NI, respectively (moderate-to-high heterogeneity, I^2^: 58–85%). Consensus-based algorithm definitions of sarcopenia, integrating low muscle mass and low levels of muscular strength and/or physical performance, lowered the prevalence (22%) and heterogeneity (I^2^ < 50%). They also increased the predictive values with RRs ranging from 2.31 (OS) to 3.52 (POC). PS among cancer patients is prevalent and strongly associated with poor outcomes during cancer treatment, especially when considering a consensus-based algorithm approach.

## 1. Introduction

Since its first definition, introduced in 1989 as an age-associated loss of muscle mass, the definition of sarcopenia has been gradually refined [1]. In 2010, 2011, and 2014, the European Working Group On Sarcopenia (namely EWGOS 1), the International Working Group on Sarcopenia (IWGS), and the Asian Working Group on Sarcopenia (AWGS 1) agreed to define sarcopenia as a syndrome characterized by an age-related loss of skeletal muscle mass (quantitatively assessed by the skeletal muscle index (SMI) or appendicular skeletal muscle mass (ASM) using CT scan, and bioelectrical impedance analysis (BIA) or dual-energy X-ray absorptiometry (DXA) methods), and by loss of function (loss of muscle strength and/or physical performance) using a screening-based approach targeting gait speed measurement [2,3,4]. In 2019, the AWGS updated the threshold values of the operational criteria (then termed AWGS 2). The EWGOS definition was also updated (EWGOS 2), now defining sarcopenia as a muscle disease characterized by the association of low levels of muscle strength (handgrip strength) and low muscle mass, with low physical performance (typically slow gait speed) becoming an indicator of severity [5,6]. In addition, the condition does not affect solely older adults ≥65 years and it has now been recognized that sarcopenia can begin earlier in life. In particular, sarcopenia is considered as primary or age-related, and as secondary when a specific cause (mainly driven by inflammatory processes) is evidenced (Table A1).

Cancer is frequently considered to be a major cause of secondary sarcopenia. In our previous systematic review including 35 observational studies or clinical trials and 6894 patients with cancer before 2016, sarcopenia concerned 38.6% of patients before cancer treatment [7]. We found that pre-therapeutic sarcopenia (PS) was associated with poor survival rates, post-operative complications and chemotherapy-related toxicities during cancer treatment, but with a strong between-study heterogeneity with respect to cancer site or extension and the definitions of sarcopenia. Since this review, with the arrival of immune-therapies and an update in the consensuses, there has been a considerable increase in the number of additional studies among cancer patients, but no comprehensive analysis has been conducted to date.

We therefore aimed to update our previous systematic review and to decipher heterogeneity in the prevalence of PS and its predictive values for overall survival, progression-free survival, post-operative complications, treatment-related toxicities, disability, and nosocomial infections among cancer patients using a meta-analysis including research published before 2022.

## 2. Materials and Methods

We followed the recommendations of the preferred reporting items for systematic reviews and meta-analyses (PRISMA) method for reporting this systematic review with meta-analysis [8]. The protocol was registered on 6 February 2023 and is available on the OSF platform: https://doi.org/10.17605/OSF.IO/H7PUZ, accessed on 13 February 2023.

### 2.1. Information Sources

This meta-analysis was based on a systematic, comprehensive search on MEDLINE via PubMed for articles published in English or French from 31 March 2016 to 31 December 2021. Due to a considerable increase in the number of studies addressing sarcopenia among cancer patients, we chose only to consult the PubMed database. The following research algorithm was used: sarcopenia AND (cancer OR tumors OR malignancies) AND (death OR overall survival OR progression-free survival OR relapse OR chemotherapy OR targeted therapy OR radiotherapy OR hormonal therapy OR surgery OR immunotherapy OR toxicity OR disability OR infection) AND human NOT review NOT letter. All articles retrieved from our previous systematic review were also included [7].

### 2.2. Search Strategy

For this meta-analysis, the following issues were addressed:

(a) What is the most commonly encountered definition of PS among patients with cancer?
(b) What is the pooled prevalence of PS among patients with cancer, and what is the prevalence according to the definition of sarcopenia?
(c) What are the mean differences in muscle strength (i.e., grip-strength) and physical performance (i.e., gait speed) between sarcopenic and non-sarcopenic groups of patients with cancer?
(d) What is the predictive value of PS for overall survival (OS) and progression-free survival (PFS) among patients with cancer?
(e) What is the predictive value of PS for severe post-operative complications (POC) among patients with cancer?
(f) What is the predictive value of PS for severe treatment-related toxicities and/or dose-limiting toxicities (TOX) among patients with cancer?
(g) What is the predictive value of PS for disability and nosocomial infections (NI) among patients with cancer?

To answer these questions, we pre-defined eligibility criteria for the articles: *patients* (adults 18 y and over with cancer), *intervention* (pre-therapeutic sarcopenia assessed using a consensual measurement), *comparator* (sarcopenia vs. no sarcopenia), *outcomes* (prevalence of sarcopenia, OS and PFS, severe post-operative complications defined as a Clavien–Dindo scale score ≥ 3a, ≥grade 3 treatment-related toxicities (CTCAE) and/or dose-limiting toxicities, disability defined in terms of an activities of daily living score (ADL) of ≤5/6, and nosocomial infections defined as hospital-acquired infections), and *study design* (clinical trials, prospective or retrospective studies with consecutive inclusions) (PICOS) criteria.

### 2.3. Selection Process

Articles meeting the eligibility criteria were first selected on the basis of titles and abstracts (FP) then on the basis of perusal of the full text by 5 independent groups (PBR/MF, ALC/CM, SM/RG, FP/JP, EL/ZapT, and EP/AN/MP). The term sarcopenia was to be clearly defined in the articles. If several articles reported similar results, only the article with the most complete information was retained. Duplicates were screened for and removed. Disagreements were resolved by consensus in each reviewing group.

### 2.4. Data Collection

The data recorded included publication date, country, study design, follow-up time, number of patients, number of men and women, cancer site, cancer extension (classified as local, locally advanced, or metastatic), treatment modes, mean or median age at inclusion, the definition of sarcopenia used (low muscle mass quantity only or consensus-based algorithm), cut-off values for quantitative muscle mass indices (arm muscle area (AMA, cm^2^), ASM (kg/m^2^), psoas muscle index (PMI, cm^2^/m^2^), SMI (cm^2^/m^2^) or total psoas area (TPA, cm^2^)), muscle strength assessed by handgrip-strength (kg), physical performance assessed by gait speed (m/s), number of sarcopenic patients, number of sarcopenic men and women, number of sarcopenic patients with a body mass index ≥30 kg/m^2^ (i.e., sarcopenic obesity), and finally the outcomes associated with either the PS values (%) or the hazard ratios or the odds ratios.

### 2.5. Meta-Analysis Endpoints

The primary endpoint was the pooled prevalence of PS among cancer patients.

The secondary endpoints were: mean differences in handgrip strength (kg) and gait speed (m/s) between sarcopenic and non-sarcopenic patients; OS; PFS; grade ≥ 3a post-operative complications (Clavien-Dindo scale); grade ≥ 3 treatment-related toxicities (CTCAE) and/or dose-limiting toxicities; ADL-score ≤ 5/6; and hospital-acquired infections.

### 2.6. Quality Assessment

We used the Newcastle–Ottawa quality assessment scale (NOS) designed for cohort studies which was the case for all patients, even for those recruited from RCTs [9]. Based on a risk-of-bias assessment, this scale rates the quality of studies with scores ranging from 0 to 9. The quality of the studies was classified as good (≥7), fair (4–6), or poor (0–3).

### 2.7. Effect Measures

The prevalence of PS was summarized as a pooled prevalence with 95% confidence interval (95% CI) using logit transformation.

Handgrip strength and gait speed were summarized as a pooled mean difference (MD) with 95% CI with reference to non-sarcopenic patients using the inverse variance method.

OS and PFS were summarized as a pooled risk ratio (RR) with 95% CI with reference to non-sarcopenic patients using the inverse variance method.

The remaining outcomes were summarized as a pooled RR with 95% CI with reference to non-sarcopenic patients using the Mantel–Haenszel method.

### 2.8. Synthesis Method

The data were analyzed using R statistical software (version 4.1.0; R Foundation for Statistical Computing, Vienna, Austria; http://www.r-project.org, accessed on 1 September 2022). All tests were 2-sided and statistical significance was set at *p* < 0.05.

Regarding the study characteristics, categorical variables were summarized as the numbers (%), and continuous variables were summarized as the means ± standard deviation (SD) or medians [Q1–Q3] as appropriate. The studies were described in descending order according to their publication date.

To detect a non-linear relationship between sarcopenia prevalence and the muscle mass indices (using reported cut-off values), we used a non-parametric regression via smoothing splines when possible.

On the basis of the selected articles, and given that between-study heterogeneity was expected, we performed a meta-analysis with random-effect models (with the package “meta”) to assess the prevalence of PS, the mean difference in muscle strength and physical performance indices (grip strength and gait speed), and the predictive value of PS for OS, PFS, Clavien–Dindo scale ≥grade 3 for POC, ≥grade-3 for TOX, disability (ADL score ≤ 5/6), and NI among cancer patients. As there was a single study addressing disability, we did not conduct a meta-analysis on this outcome. With regard to the prevalence of PS (first endpoint), we first ran a funnel plot to detect graphical asymmetry. Statistically, the funnel plot asymmetry was assessed using the Peters’ test, which is appropriate for meta-analyses of single proportion. We addressed the heterogeneity of the study results using the I^2^ indicator and the Cochran’s Q test. I^2^ values of 0%, 25%, 50%, and 75% were considered to indicate none, low, moderate, and high heterogeneity, respectively. A *p* value ≤ 0.05 in the Q test indicated a significant heterogeneity. Due to the heterogeneous nature of sarcopenia and the variety of the contexts assessed, we anticipated the need for subgroup analyses according to a sensitivity analysis, excluding studies over the 95% confidence interval from the funnel plot, according to the following: study quality (good, fair or poor), the mean or median age at inclusion classified as < or ≥65 y, sex, BMI (< or ≥30 kg/m^2^), cancer site, cancer extension, treatment mode, definition of sarcopenia (low muscle mass quantity only or consensus-based algorithm), and the cut-off values for muscle mass indices. We also considered post hoc subgroup analyses according to the publication date (2008–2012, 2013–2017, and 2018–2022), the number of patients included (<100, 100–200, 200–400, and ≥400), and world regions (Asian vs. non-Asian).

To decipher the factors that could explain heterogeneity, we then ran a multivariate meta-regression with a mixed-effect model for the first endpoint (prevalence). Factors (study groups) yielding *p* values under 0.20 in the univariate analysis were considered for inclusion in the multivariate analysis. A backward selection process of the highest *p* values was performed to retain the final multivariate model.

## 3. Results

### 3.1. Study Selection and Quality Rating of the Studies Included

**Figure A1:**
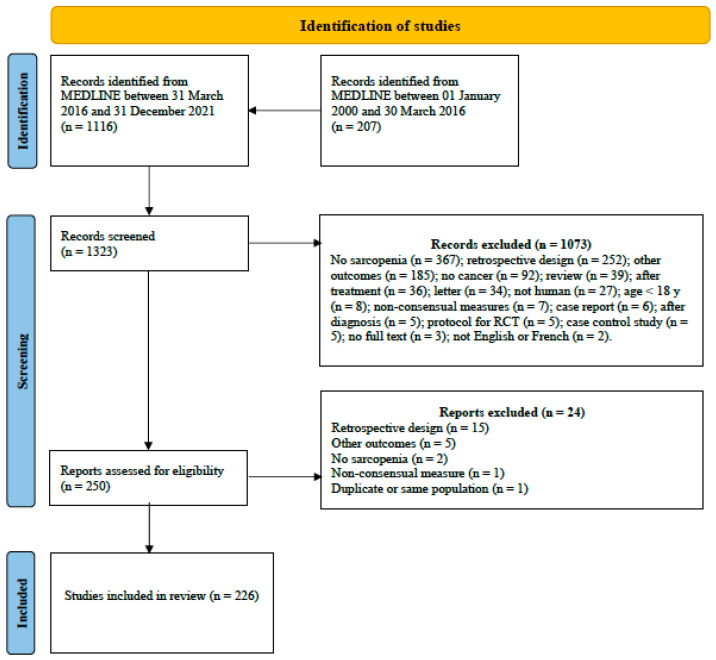
Flaw chart of the meta-analysis.

As of 31 December 2021, including the final publications in 2022, the comprehensive search yielded 1318 articles potentially eligible for this review (Figure A1). After excluding non-eligible articles, 226 remained for review and meta-analysis, dated from 2008 to 2022 as follows: 5 in 2022 [10,11,12,13,14], 60 in 2021 [15,16,17,18,19,20,21,22,23,24,25,26,27,28,29,30,31,32,33,34,35,36,37,38,39,40,41,42,43,44,45,46,47,48,49,50,51,52,53,54,55,56,57,58,59,60,61,62,63,64,65,66,67,68,69,70,71,72,73,74], 36 in 2020 [75,76,77,78,79,80,81,82,83,84,85,86,87,88,89,90,91,92,93,94,95,96,97,98,99,100,101,102,103,104,105,106,107,108,109,110], 31 in 2019 [111,112,113,114,115,116,117,118,119,120,121,122,123,124,125,126,127,128,129,130,131,132,133,134,135,136,137,138,139,140,141], 29 in 2018 [142,143,144,145,146,147,148,149,150,151,152,153,154,155,156,157,158,159,160,161,162,163,164,165,166,167,168,169,170], 18 in 2017 [171,172,173,174,175,176,177,178,179,180,181,182,183,184,185,186,187], 15 in 2016 [188,189,190,191,192,193,194,195,196,197,198,199,200,201,202], 15 in 2015 [203,204,205,206,207,208,209,210,211,212,213,214,215,216,217], 2 in 2014 [218,219], 5 in 2013 [220,221,222,223,224], 6 in 2012 [225,226,227,228,229,230], none in 2011, 1 in 2010 [231], 2 in 2009 [232,233], and 1 in 2008 [234] (Table 1).

According to the NOS score, 151/226 studies (67%) were rated as good quality (NOS ≥ 7) meaning there was a low risk of bias (Table 1).

### 3.2. Patient and Study Characteristics

In all, 65,936 patients were included in this meta-analysis with sample sizes ranging from 16 to 6447 patients (Table 1). Overall, 118 studies were retrospective, 95 were prospective, and 13 were clinical trials. The studies were mainly from Asia, Europe, and North-America, while only two studies were from Africa [75,101]. The follow-up time ranged from 0 to 200 months. The mean or median age at inclusion ranged from 45.7 to 85 y. Thirty-three per cent (17,295/65,936) of the patients had a mean or median age at inclusion ≥65 years. A total of 419/65,936 patients had a mean or median age at inclusion ≥75 years. Most of the studies also included patients younger than 65 years (64%, 30,691/47,986 patients), Asians (51%, 33,453/65,936), men (66%, 30,424/46,265), and with a body mass index < 30 kg/m^2^ (69.5%, 2628/8627). The studies mainly included cancers in various sites (22%, 14,600/65,936), gastric (20.5%, 13,513/65,936), or colorectal (17%, 11,419/65,936), and with various extensions (82%, 54,269/65,936). The treatment modes observed were mainly surgery (61%, 40,486/65,936), chemotherapy (6%, 4169/65,936), immune therapy (1%, 909/65,936), and targeted therapy (1%, 634/65,936).

### 3.3. Definition of Sarcopenia among Cancer Patients

Sarcopenia was mainly defined from muscle mass measurement only (190/226 studies, 84%), from CT scan (n = 178), BIA (n = 11), or DXA (n = 1) (Table 1). Sixteen studies did not specify the muscle mass index used [43,45,68,85,86,95,96,120,122,123,139,141,147,148,186,207]. Of the 210 remaining studies, regardless of the sarcopenia definition used, the SMI by CT scan at lumbar three was the main muscle mass quantity index used (171/210, 81.5%) followed by the ASM (21/210, 10%), the PMI (13/210, 6%), the TPA (4/210 studies, 2%), and the AMA (1/226 studies, 0.5%). The SMI cut-off values ranged from 29.0 to 48.4 cm^2^/m^2^ (37 thresholds in all) for women (median [Q1–Q3] = 38.5 [37.5–41.0]), and from 36.0 to 68.9 cm^2^/m^2^ (43 thresholds in all) for men (median [Q1–Q3] = 47.3 [43.0–52.4]).

From 2015 to the present, of the 36 studies applying a consensus-based algorithm definition of sarcopenia, 17, 9, 6, and 2 studies respectively used the AWGS1, the EWGOS2, the EWGOS1, and the AWGS2 guidelines. The IWGS was not used.

### 3.4. PS Is Prevalent among Cancer Patients

**Figure A2:**
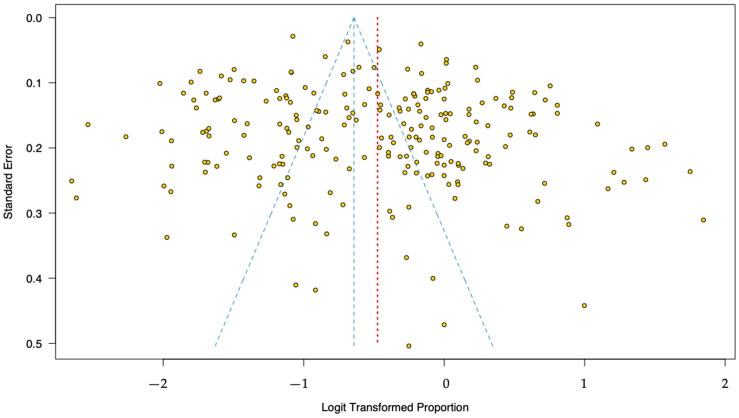
Funnel plot of the prevalence of pre-therapeutic sarcopenia among cancer patients (*p* value for asymmetry [Peters’ test] < 0.0001). Gold points = studies; blue dash lines = common effect with 95% CI; red dash lines = random effect.

All the studies were used to assess the prevalence. The pooled prevalence of PS among cancer patients was 38.0% (95% CI: 36.0–41.0) with a high between-study heterogeneity (I^2^ = 97%) (Table 2). Figure A2 shows a significant funnel plot asymmetry (*p* < 0.0001).

Although the sensitivity analysis excluding studies over the 95% confidence interval from the funnel plot (n = 137 studies) led to less heterogeneity (I^2^ = 66%), the 40.5% prevalence was not significantly different (*p* = 0.11).

The prevalence was significantly lower (*p* < 0.01) for consensus-based algorithm definitions of sarcopenia (22.0%) than for definitions based on muscle mass measures only (42.0%).

**Figure 1:**
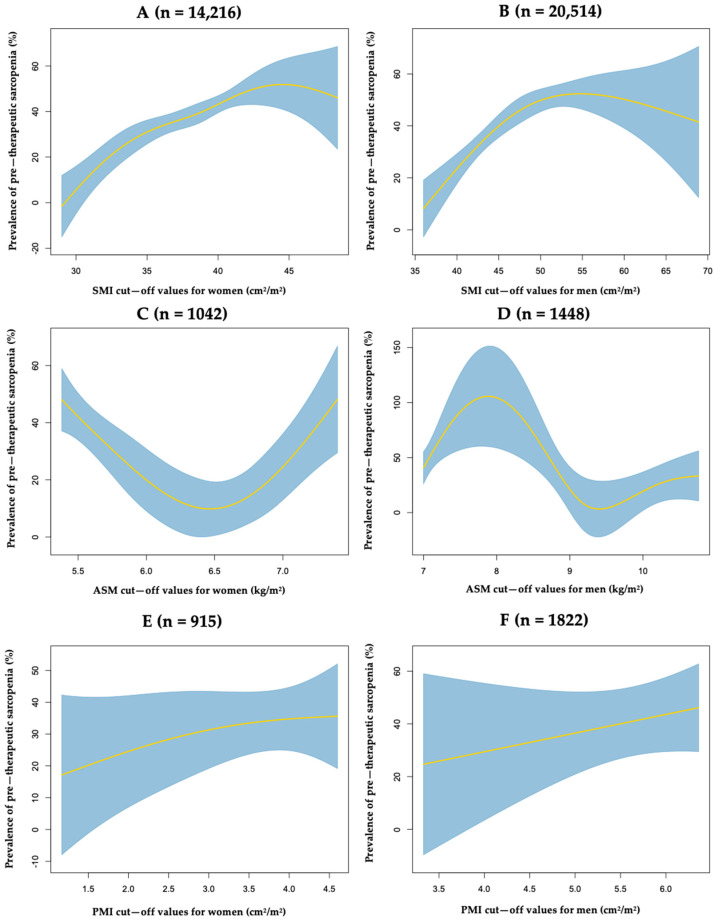
Non-parametric regression via smoothing splines for assessing the relationship between prevalence of pre-therapeutic sarcopenia and cut-off values of SMI (**A**,**B**), ASM (**C**,**D**), or PMI (**E**,**F**) in women and men with cancer, respectively.

Using the muscle mass measurement-based definition only, the prevalence differed significantly (*p* < 0.01), ranging from 12.0% (AMA) to 40.0% (SMI). Above the SMI medians of 38.5 cm^2^/m^2^ and 47.3 cm^2^/m^2^ for women and men, respectively, the prevalence was 47.0% and 52.0%. Figure 1 shows the smoothing splines for the relationship between the prevalence of sarcopenia and muscle mass index measures for women and men (SMI, ASM, and PMI). Regarding SMI, up to the third quartiles of 41.0 cm^2^/m^2^ and 52.4 cm^2^/m^2^ for women and men, respectively, the association was linear with a tight confidence interval. Regarding ASM, the association was paradoxical for both women and men. For PMI, the association was strictly linear for women and men, but it resulted in a large confidence interval.

Depending on the cancer site, the prevalence varied very significantly from 24.0% (gastric) to 79.0% (small cell lung). In relation to cancer extension, the prevalence varied significantly from 39.0% (local) to 46.0% (metastatic). According to the treatment mode, the pre-therapeutic prevalence of sarcopenia varied significantly from 33.0% (surgery) to 68% (intra-arterial infusion for hepatocellular carcinoma). In the 26 studies that reported prevalence according to BMI, the prevalence of sarcopenic obesity was significantly lower (*p* < 0.01) (19.0%) than for non-sarcopenic obesity (39.0%). The prevalence did not differ significantly with study quality, the year of publication, the world region, the age-threshold of 65 years at inclusion, or sex.

In multivariate meta-regression, consensus-based algorithm definitions of sarcopenia (as opposed to loss of muscle mass only), a sample size ≥ 400, and SMI based on CT scan cut-off values for women (not for men) as continuous variables were independently and significantly associated with the prevalence results (Table A2).

### 3.5. Muscle Strength and Physical Performance among Cancer Patients with Sarcopenia

**Figure 2:**
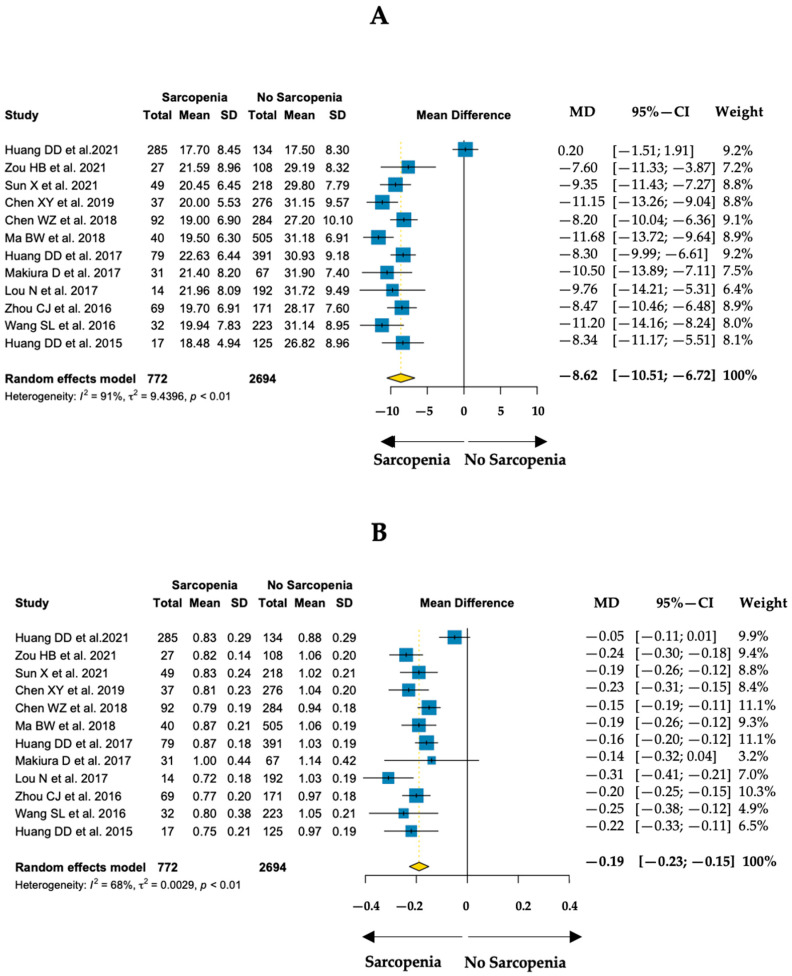
Pooled mean differences (MD) of handgrip strength (**A**, kg) and gait speed (**B**, m/s) between sarcopenic and non-sarcopenic groups among cancer patients [26,51,58,111,138,145,169,177,179,187,206,216].

Twelve studies including 3466 patients provided data on muscle strength and physical performance. Handgrip strength values among sarcopenic patients ranged from 17.7 to 22.6 kg and gait speed values among sarcopenic patients ranged from 0.72 to 1.00 m/s [26,51,58,111,138,145,169,177,179,187,206,216]. Figure 2 summarizes the mean differences in handgrip strength and gait speed between sarcopenic and non-sarcopenic patients.

For handgrip strength, the pooled mean difference was −8.62 kg with a high between-study heterogeneity (I^2^ = 91%). Regarding gait speed, the pooled mean difference was −0.19 m/s with a moderate between-study heterogeneity (I^2^ = 68%).

### 3.6. Pre-Therapeutic Sarcopenia Is Associated with OS and PFS among Cancer Patients

**Figure A3:**
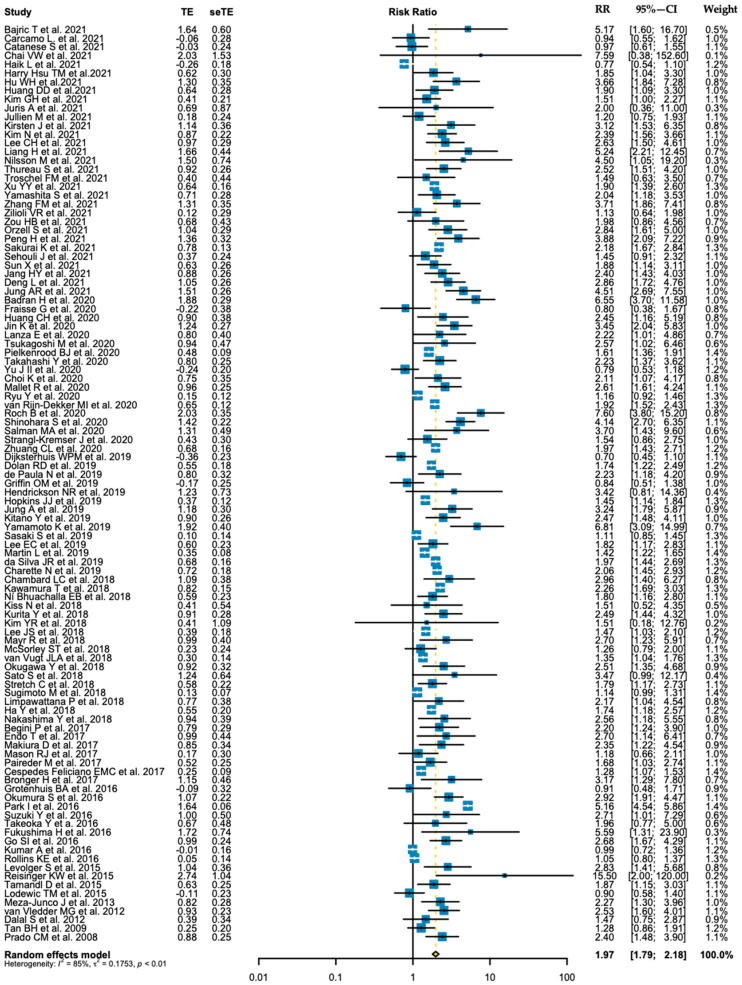
Pooled relative risk (RR) of the predictive value of pre-therapeutic sarcopenia on overall survival among cancer patients [14,15,16,17,18,23,24,25,26,28,30,33,34,36,37,39,42,43,47,48,49,50,51,52,55,56,58,61,65,67,70,72,75,77,79,80,81,83,89,91,93,94,96,98,100,101,102,103,104,107,108,112,113,114,115,116,117,119,125,130,132,133,135,136,144,146,147,148,149,150,153,155,159,161,162,163,165,167,168,169,170,171,174,181,184,189,191,193,194,195,196,197,198,200,209,210,211,214,224,229,230,232,234].

Based on 101 studies including 28,995 patients, we found a strong, significant association between pre-therapeutic sarcopenia and OS with a pooled RR of 1.97 [1.79–2.17] and with a high between-study heterogeneity (I^2^ = 85%, *p* < 0.01). Subgroup analyses are presented in Table 3 and Figure A3, showing a reduction in heterogeneity. The effect measure differed significantly (*p* < 0.01) according to sample size, world region, cancer site, and muscle mass index, while no significant differences were found for sensitivity analysis, study quality, year of publication, age threshold of 65 years at inclusion, cancer extension, treatment mode, and definition of sarcopenia. When low between-study heterogeneity was envisaged (i.e., I^2^ < 50%), the greatest effects were associated with the PMI-based muscle index (RR = 2.76 [2.21–3.43]), bile duct cancers (RR = 2.71 [1.87–3.92]), and the consensus-based algorithm definitions of sarcopenia (RR = 2.31 [1.97–2.72]).

**Figure A4:**
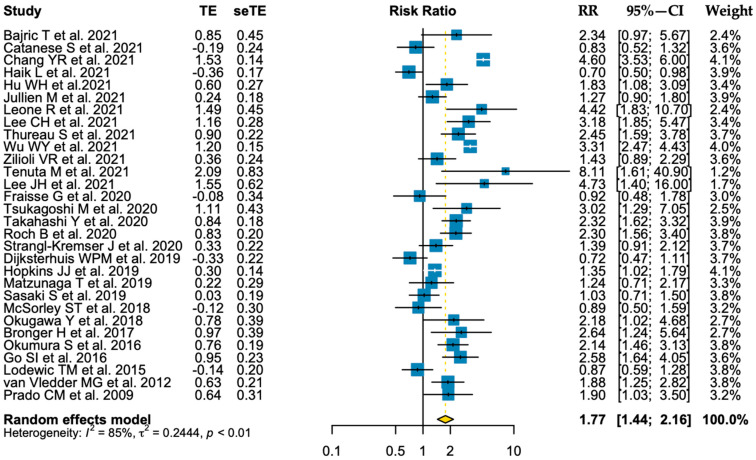
Pooled relative risk (RR) of the predictive value of pre-therapeutic sarcopenia on progression-free survival among cancer patients [15,17,19,23,25,31,35,36,42,46,50,62,63,72,77,81,98,102,112,116,122,130,153,159,184,191,197,214,229,233].

For 29 studies including 6546 patients, we found a strong and significant association between pre-therapeutic sarcopenia and PFS with a pooled RR of 1.76 [1.44–2.16] and a high between-study heterogeneity (I^2^ = 85%, *p* < 0.01). Subgroup analyses are presented in Table 4 and Figure A4, showing a reduction in heterogeneity. The effect measure differed significantly (*p* < 0.01) according to the world region, cancer site, sarcopenia definition, and muscle mass index used, while no significant differences were found for sensitivity analysis, study quality, year of publication, sample size, age threshold of 65 years at inclusion, cancer extension, or treatment mode. When low between-study heterogeneity was envisaged (i.e., I^2^ < 50%), the most marked effects were associated with the consensus-based algorithm definitions of sarcopenia (RR = 3.59 [2.17–5.92]) and non-small cell lung cancer (RR = 2.43 [1.90–3.12]).

### 3.7. Pre-Therapeutic Sarcopenia Is Predictive of Severe Postoperative Complications among Cancer Patients

**Figure A5:**
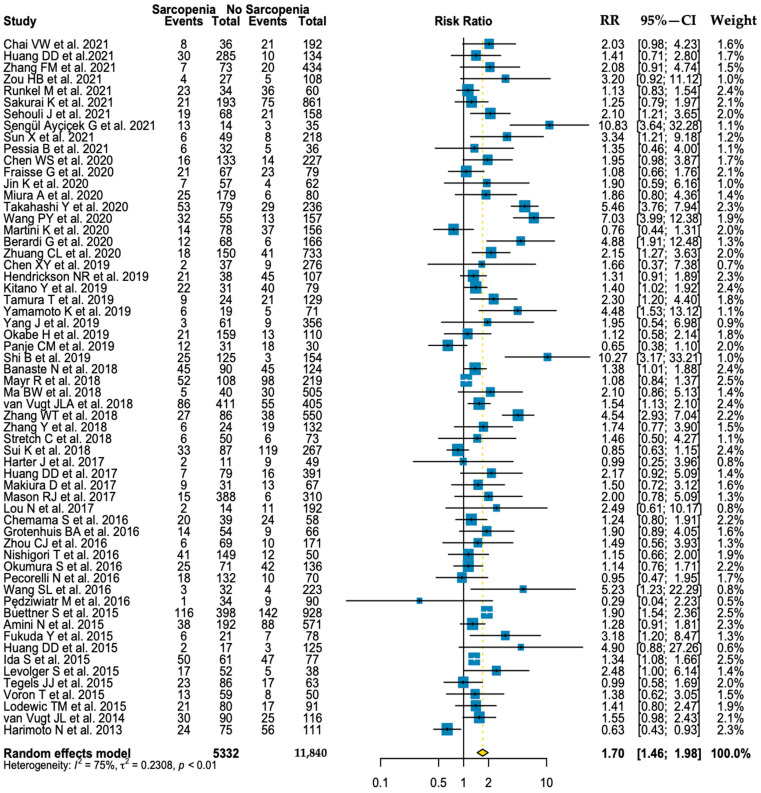
Pooled relative risk (RR) of the predictive value of pre-therapeutic sarcopenia on severe post-operative complications among cancer patients [18,26,49,51,54,55,56,57,58,59,70,71,72,76,77,84,85,86,103,104,111,119,123,125,126,127,129,131,138,143,150,155,157,158,162,164,169,170,175,177,179,184,187,188,190,191,192,199,202,203,205,206,207,209,212,213,214,216,217,220].

Based on 56 studies including 17,172 patients, we found a strong and significant association between pre-therapeutic sarcopenia and severe post-operative complications, with a pooled RR of 2.70 [2.33–3.12] involving a moderate heterogeneity (I^2^ = 72%). Subgroup analyses are presented in Table 5 and Figure A5, showing a reduction in heterogeneity. The effect measure differed significantly (*p* < 0.01) according to sensitivity analysis, year of publication, sample size, world region, cancer site, and sarcopenia definition, while no significant differences were found for study quality, the age threshold of 65 years at inclusion, cancer extension, or muscle mass index. When low between-study heterogeneity was envisaged (i.e., I^2^ < 50%), the most marked effects were associated with the consensus-based algorithm definitions of sarcopenia (RR = 3.62 [2.79–4.69]) and gastric cancer (RR = 3.09 [2.42–3.93]).

### 3.8. Pre-Therapeutic Sarcopenia Is Predictive of Severe Treatment-Related Toxicity and/or Dose-Limiting Toxicity among Cancer Patients

**Figure A6:**
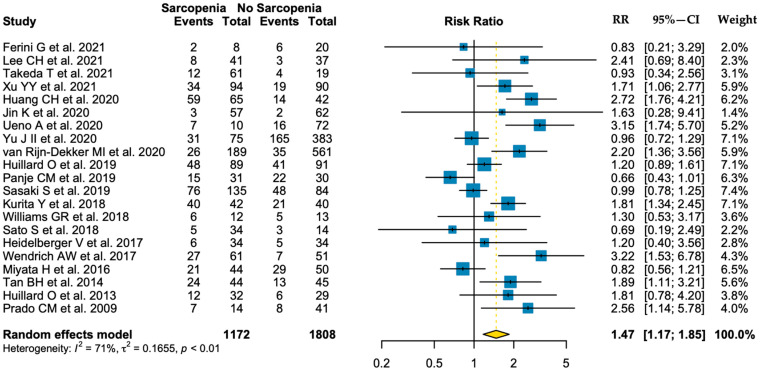
Pooled relative risk (RR) of the predictive value of pre-therapeutic sarcopenia on severe treatment-related toxicity and/or dose limiting toxicity among cancer patients [22,36,40,47,70,79,82,89,96,118,129,130,136,156,161,176,183,186,215,221,233].

Based on 19 studies including 2980 patients, we found a significant association between pre-therapeutic sarcopenia and severe treatment-related toxicities and/or dose-limiting toxicities, with a pooled RR of 1.47 [1.17–1.84] involving a moderate heterogeneity (I^2^ = 71%). Subgroup analyses are presented in Table 6 and Figure A6, showing a reduction in heterogeneity. The effect measure differed significantly (*p* < 0.01) according to study quality, sample size, cancer site, cancer extension, and definition of sarcopenia, while no significant differences were found for sensitivity analysis, year of publication, world region, age threshold of 65 years at inclusion, or treatment mode. When low between-study heterogeneity was envisaged (i.e., I^2^ < 50%), the most marked effects were associated with breast cancer (RR = 2.93 [1.82–4.73]), head and neck cancer (RR = 2.47 [1.65–3.69]), chemotherapy (RR = 1.98 [1.55–2.54]), and targeted therapy (RR = 1.63 [1.05–2.54]).

### 3.9. Pre-Therapeutic Sarcopenia Is Associated with Disability among Cancer Patients

Only one study including 131 patients was found on the association between pre-therapeutic sarcopenia and disability (ADL ≤ 5/6) [160]. In this single-center prospective study including 40.5% of patients aged ≥ 75 years with cancers in various sites and with different extensions, baseline disability was noted for 30.5% of the patients. Compared to normal muscle mass and non-severe sarcopenia, severe sarcopenia is defined according to the EWGOS1 by low muscle mass (CT scan-based SMI), and both low handgrip strength and slow gait speed were significantly (*p* < 0.001) associated with disability (90% vs. 26% of patients) in univariate analysis.

### 3.10. Pre-Therapeutic Sarcopenia Is Predictive of Nosocomial Infections among Cancer Patients

**Figure A7:**
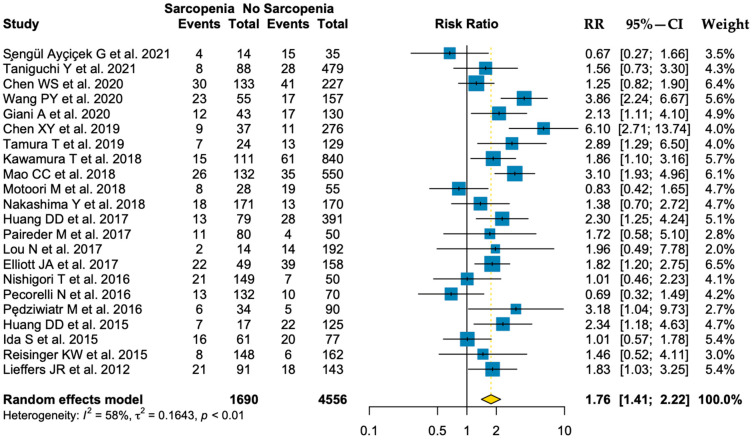
Pooled relative risk (RR) of the predictive value of pre-therapeutic sarcopenia on nosocomial infections among cancer patients [57,64,76,84,95,111,123,146,151,152,168,177,179,182,190,192,199,206,207,210,225,235].

Based on 22 studies including 6246 patients, we found a strong, significant association between pre-therapeutic sarcopenia and nosocomial infections with a pooled RR of 1.76 [1.41–2.22] and moderate heterogeneity (I^2^ = 58%). Subgroup analyses are presented in Table 7 and Figure A7, showing a reduction in heterogeneity. The effect measure differed significantly (*p* < 0.01) according to sensitivity analysis, sample size, age threshold of 65 years at inclusion, cancer site, and definition of sarcopenia, while no significant differences were found for study quality, year of publication, world region, cancer extension, treatment mode, or muscle mass index. When low between-study heterogeneity was envisaged (i.e., I^2^ < 50%), the most marked effects were associated with gastric cancer (RR = 2.55 [1.88–3.46]) and a sample size ≥ 400 (RR = 2.26 [1.66–3.07]).

## 4. Discussion

In this meta-analysis including 226 articles and 65,936 patients with various cancers, various extensions, and various treatment modes, PS was mainly defined as a loss of muscle mass using the SMI on CT scan-based assessment. PS was highly prevalent and was strongly associated with OS, PFS, POC, TOX, and NI during cancer treatment, with pooled relative risks ranging from 1.50 (toxicities) to 2.70 (post-operative complications).

To date, and despite successive sarcopenia consensus-based definitions of sarcopenia provided since 2010, the definition of sarcopenia mainly relies only on loss of muscle mass quantity among cancer patients. The standardized use of CT scans in pre-therapeutic oncological settings probably explains this. Unlike the ASM and the PMI indices, the SMI muscle mass index was linearly associated with the prevalence of sarcopenia for both women and men and had the tightest confidence interval, suggesting that it is probably the most suitable index for the quantification of muscle mass. However, homogeneous optimal cut-off thresholds remain to be clarified in the oncological setting.

As expected, we found great heterogeneity for all endpoints addressed here. Consistent with our previous review, the pooled prevalence of pre-therapeutic sarcopenia concerned 38% of cancer patients [7]. Using a multivariate meta-regression, we were able to identify sources of between-study heterogeneity as follows: consensus-based algorithm definitions of sarcopenia (as opposed to loss of muscle mass only), a powerful sample size (≥400), and the cut-off values of CT scan-based SMI for women (not for men) as continuous variables were independently and significantly associated with the prevalence results for pre-therapeutic sarcopenia. With respect to the prevalence results according to cancer localisation, our results require caution given the impact of the definition used. Strikingly, compared with definitions based on loss of muscle mass only (SMI), consensus-based algorithm definitions of sarcopenia reduced the prevalence significantly (42% vs. 22%), decreasing heterogeneity and increasing the predictive value for OS (RR = 1.85 vs. 2.31), PFS (RR = 1.61 vs. 3.59), post-operative complications (RR = 1.48 vs. 3.62), and nosocomial infections (RR = 1.85 vs. 2.49). This discrepancy could be explained by the additional criteria used for consensus algorithms, which consider both loss of muscle strength and/or physical performance and muscle mass. Indeed, it is known that grip strength and gait speed are independent factors associated with survival among cancer patients [236].

Surprisingly, except for nosocomial infections, we did not identify any significant difference for prevalence of sarcopenia, OS, PFS, post-operative complications, or severe treatment-related toxicities according to the age threshold of 65. This result highlights the leading role played by cancer (mainly due to cancer-related inflammatory processes) rather than age alone in promoting sarcopenia (namely secondary sarcopenia) and its clinical impact on adverse outcomes [6].

To our knowledge, although it was not performed on individual data, this is the largest and most powerful meta-analysis on this topic. It contains a stringent methodology, bringing together oncologists, geriatricians, and methodologists using data from many countries in numerous cancer settings, enabling us to provide a comprehensive up-to-date review of sarcopenia prevalence and its clinical impact in the course of cancer treatment. In particular, in a cancer setting we were able to highlight an association between pre-therapeutic sarcopenia and PFS on the one hand, and between pre-therapeutic sarcopenia and nosocomial infections on the other, subjects that have been studied infrequently to date. However, there are still insufficient data to provide a synthesis regarding the association between pre-therapeutic sarcopenia and disability.

On the basis of the findings of our meta-analysis, there clearly is an urgent need to agree on an operational definition of sarcopenia in oncological settings to improve study comparability. Given both the high prevalence and the strong clinical impact of pre-therapeutic sarcopenia during cancer treatment, we suggest that its detection should occur as early as possible. In agreement with the EWGOS 2 consensus, we support the use of the simple SARC-F (strength, assistance with walking, rise from a chair, climb stairs, and falls) screening tool, which has been previously validated in older cancer patients [237]. The early detection of sarcopenia can help to initiate early muscle rehabilitation combining protein supplementation and resistance exercise training in order to improve the healthcare trajectories during cancer treatment [238]. Finally, we encourage the use of sarcopenia as a stratification variable in the development of future clinical trial designs in oncology.

## 5. Conclusions

Using the findings of the largest and the most powerful meta-analysis on this topic to date, we conclude that pre-therapeutic sarcopenia among cancer patients, mainly defined as a loss of muscle mass quantity, is prevalent and strongly associated with OS, PFS, severe post-operative complications, severe treatment-related toxicities and/or dose-limiting toxicities, and nosocomial infections. We stress the need to agree on a consensual definition of sarcopenia in oncological settings.

## Figures and Tables

**Table 1:** Study characteristics.

Reference	Asia (Y/N)	Recruitment RCT/P/RP	Follow-Up (m)	Patients			Site	Extension	Treatment	Mean or Median Age (y)	Definition of Sarcopenia/Muscle Mass Index	Sarcopenia			Over the 95% CI (Y/N)	NOS G/F/P
				Total	M	F						Total	M	F		
Takagi A et al., 2022 [10]	Y	P	1.0	114	74	40	Various	Various	Various	68.4	CT scan/SMI	57	NA	NA	N	G
Lu JL et al., 2022 [11]	Y	P	0.0	260	196	64	Gastric	Locally advanced	Surgery	62.4	EWGOS 2/ASM	41	22	19	Y	G
Deluche E et al., 2022 [12]	N	P	1.0	139	2	137	Breast	Metastatic	Various	61.2	EWGOS 1/SMI	41	1	40	N	F
Tagliafico AS et al., 2022 [13]	N	P	200.0	74	37	37	Myeloma	Metastatic	Chemotherapy	60.8	CT scan/SMI	18	6	12	Y	G
Orzell S et al., 2022 [14]	N	P	72.0	251	191	58	Head and neck	Various	Surgery	67.4	EWGOS 2/SMI	39	21	18	Y	G
Bajric T et al., 2021 [15]	N	RP	63.6	355	135	220	Colorectal	Metastatic	Surgery	68.0	CT scan/SMI	78	65	13	Y	F
Cárcamo L. et al., 2021 [16]	N	RP	72.0	359	193	166	Colorectal	Various	Surgery	64.0	CT scan/SMI	85	NA	NA	Y	G
Catanese S et al., 2021 [17]	N	RP	87.6	78	56	22	Gastric	Metastatic	Various	67.0	CT scan/SMI	34	22	12	N	G
Chai VW et al., 2021 [18]	N	RP	12.0	228	139	89	Colorectal	Various	Surgery	69.0	CT scan/SMI	36	24	12	Y	G
Chang YR et al., 2021 [19]	Y	RP	141.6	109	63	46	Sarcoma	Metastatic	Targeted therapy	61.0	CT scan/PMI	25	NA	NA	Y	F
Chen HW et al., 2021 [20]	Y	RP	86.4	163	NA	NA	Urothelial	Various	Surgery	64.3	CT scan/SMI	132	NA	NA	Y	F
Daffrè E et al., 2021 [21]	N	RP	60.0	238	169	69	Lung NSC	Various	Surgery	63.0	CT scan/SMI	47	36	11	Y	G
Ferini G et al., 2021 [22]	N	RP	70.0	28	25	3	Urothelial	Various	Radiotherapy	85.0	CT scan/SMI	8	8	0	N	F
Haik L et al., 2021 [23]	N	RP	60.0	261	198	63	Various	Various	Immunotherapy	61.9	CT scan/SMI	122	87	35	N	F
Harry Hsu TM et al., 2021 [24]	N	P	33.6	136	63	73	Pancreas	Various	Not specified	67.0	CT scan/SMI	21	9	12	Y	F
Hu WH et al.,2021 [25]	Y	RP	80.4	114	68	46	Colorectal	Various	Surgery	63.2	CT scan/SMI	52	NA	NA	N	G
Huang DD et al.,2021 [26]	Y	P	67.2	419	282	137	Gastric	Various	Surgery	72.0	CT scan/SMI	285	208	77	Y	G
Kim J et al., 2021 [27]	Y	RP	41.0	840	526	534	Gastric	Various	Various	60.4	CT scan/SMI	119	110	9	Y	G
Kim GH et al., 2021 [28]	Y	RP	70.5	280	182	98	Gastric	Local	Surgery	82.0	CT scan/SMI	173	NA	NA	Y	G
Kawaguchi Y et al., 2021 [29]	Y	RP	60.0	256	173	83	Lung NSC	Various	Surgery	68.5	CT scan/PMI	128	89	39	Y	F
Juris A et al., 2021 [30]	N	RP	48.0	89	40	49	Various	Various	Various	57.0	CT scan/SMI	22	11	11	Y	F
Jullien M et al., 2021 [31]	N	P	36.6	656	367	289	Lymphoma	Various	Various	49.0	CT scan/SMI	225	179	46	N	G
Jalal M et al., 2021 [32]	N	RP	0.0	204	114	90	Pancreas	Locally advanced	Various	69.0	CT scan/SMI	111	41	70	Y	F
Kirsten J et al., 2021 [33]	N	P	12.0	178	109	69	Leukemia	Metastatic	Chemotherapy	58.3	EWGOS1	49	42	7	Y	G
Kim N et al., 2021 [34]	Y	RP	30.1	185	120	65	Gastric	Metastatic	Immunotherapy	59.0	CT scan/SMI	93	85	8	Y	G
Leone R et al., 2021 [35]	N	RP	40.0	43	15	28	Lymphoma	Various	Chemotherapy	61.0	CT scan/SMI	13	NA	NA	N	F
Lee CH et al., 2021 [36]	Y	RP	57.1	78	59	19	Kidney	Metastatic	Targeted therapy	61.6	CT scan/SMI	41	28	13	N	G
Liang H et al., 2021 [37]	Y	RP	17.7	100	93	7	Esophageal	Various	Radiotherapy	59.0	CT scan/SMI	77	74	3	Y	F
Makal GB et al., 2021 [38]	Y	RP	1.0	225	141	84	Various	Various	Surgery	58.7	CT scan/TPA	102	42	60	N	P
Nilsson M et al., 2021 [39]	N	RP	60.0	106	22	84	Anal	Various	Radiotherapy	63.8	CT scan/SMI	41	11	30	N	G
Takeda T et al., 2021 [40]	Y	RP	63.6	80	35	45	Pancreas	Metastatic	Chemotherapy	77.0	CT scan/SMI	61	25	36	Y	G
Takiguchi K et al., 2021 [41]	Y	RP	96.0	209	116	93	Colorectal	Locally advanced	Surgery	NA	CT scan/PMI	81	50	31	N	F
Thureau S et al., 2021 [42]	N	P	60.0	243	187	56	Head and neck	Various	Various	61.0	CT scan/SMI	88	NA	NA	N	G
Troschel FM et al., 2021 [43]	N	RP	96,0	367	247	120	Lung NSC	Various	Surgery	62.2	CT scan/NA	104	86	18	Y	G
Trussardi Fayh AP et al., 2021 [44]	N	P	0.0	108	51	57	Various	Various	Various	70.6	EWGOS 2/SMI	26	NA	NA	Y	F
van den Berg I et al., 2021 [45]	N	RP	60.0	754	352	306	Colorectal	Various	Surgery	NA	CT scan/NA	266	NA	NA	N	F
Wu WY et al., 2021 [46]	Y	P	67.2	648	486	162	Gastric	Various	Surgery	64.3	AWGS2/ EWGOS2/SMI	133	91	42	Y	G
Xu YY et al., 2021 [47]	Y	RP	50.0	184	141	43	Esophageal	Various	Various	62.0	CT scan/SMI	94	75	19	Y	F
Yamashita S et al., 2021 [48]	Y	RP	72.0	123	103	20	Urothelial	Various	Surgery	74.0	CT scan/SMI	48	NA	NA	N	F
Zhang FM et al., 2021 [49]	Y	P	80.0	507	367	140	Gastric	Local	Surgery	63.0	CT scan/SMI	73	53	20	Y	F
Zilioli VR et al., 2021 [50]	N	RP	144.0	154	78	76	Lymphoma	Various	Various	71.0	CT scan/SMI	66	42	24	N	G
Zou HB et al., 2021 [51]	Y	P	6.0	135	91	44	Gastric	Various	Surgery	64.0	AWGS 2/SMI	27	14	13	Y	F
Peng H et al., 2021 [52]	Y	RP	82.0	121	96	25	Esophageal	Various	Surgery	70.3	CT scan/SMI	65	52	13	Y	F
Rinninella E et al., 2021 [53]	N	RP	0.0	26	18	8	Gastric	Locally advanced	Various	63.3	CT scan/SMI	19	NA	NA	Y	F
Runkel M et al., 2021 [54]	N	RP	0.0	94	58	36	Colorectal	Metastatic	Surgery	61.4	CT scan/SMI	34	NA	NA	N	F
Sakurai K et al., 2021 [55]	Y	RP	127.0	1054	691	363	Gastric	Various	Surgery	NA	CT scan/SMI	193	117	76	Y	G
Sehouli J et al., 2021 [56]	N	P	59.0	226	0	226	Various	Various	Surgery	59.0	BIA/ASM	68	0	68	N	G
Şengül Ayçiçek G et al., 2021 [57]	N	P	0.0	49	25	24	Various	Various	Surgery	70.0	BIA/ASM	14	1	13	N	F
Sun X et al., 2021 [58]	Y	P	50.0	267	202	65	Gastric	Various	Surgery	64.8	AWGS 1/SMI	49	32	17	Y	G
Pessia B et al., 2021 [59]	N	RP	48.0	68	NA	NA	Pancreas	Local	Surgery	63.0	CT scan/SMI	32	NA	NA	N	G
Choi H et al., 2021 [60]	Y	RP	60.0	440	243	197	Lung NSC	Local	Surgery	65.0	CT scan/SMI	246	NA	NA	Y	G
Jang HY et al., 2021 [61]	Y	RP	120.0	160	120	40	Liver	Local	Surgery	55.2	CT scan/SMI	28	17	11	Y	G
Tenuta M et al., 2021 [62]	N	P	62.5	47	27	20	Lung NSC	Locally advanced	Immunotherapy	67.0	EWGOS 2/ASM	19	10	9	N	G
Lee JH et al., 2021 [63]	Y	P	36.0	70	70	0	Prostate	Metastatic	Various	66.5	CT scan/SMI	47	47	0	Y	G
Taniguchi Y et al., 2021 [64]	Y	RP	72.0	567	393	174	Gastric	Various	Surgery	NA	CT scan/PMI	88	81	7	Y	G
Deng L et al., 2021 [65]	Y	P	80.0	121	52	69	Cholangiocarcinoma	Various	Surgery	65.0	CT scan/PMI	53	NA	NA	N	G
Uemura S et al., 2021 [66]	Y	RP	60.0	69	38	31	Pancreas	Various	Chemotherapy	63.0	CT scan/SMI	33	12	21	N	F
Jung AR et al., 2021 [67]	Y	P	96.0	190	156	34	Head and neck	Various	Various	71.9	CT scan/SMI	64	56	8	N	G
Huang X et al., 2021 [68]	Y	P	3.0	82	55	27	Head and neck	Various	Chemotherapy	45.7	AWGS 1//NA	37	17	20	N	G
Regnier R et al., 2021 [69]	N	RP	3.0	82	62	20	Kidney	Locally advanced	Various	65.0	CT scan/SMI	47	39	8	Y	G
Jin K et al., 2021 [70]	Y	RP	0.0	119	59	60	Pancreas	Locally advanced	Various	60.2	CT scan/SMI	57	NA	NA	N	G
Miura A et al., 2021 [71]	Y	RP	79.6	259	155	104	Lung NSC	Various	Surgery	73.0	CT scan/PMI	179	127	52	Y	F
Takahashi Y et al., 2021 [72]	Y	RP	137.0	315	192	123	Lung NSC	Local	Surgery	70.0	CT scan/PMI	79	46	33	Y	G
Silva PB et al., 2021 [73]	N	P	0.0	71	71	0	Head and neck	Various	Not specified	66.9	EWGOS 1/ASM	32	32	0	N	F
Seror M et al., 2021 [74]	N	RP	60.0	110	92	18	Liver	Local	Surgery	67.7	CT scan/SMI	26	25	1	Y	G
Badran H et al., 2020 [75]	N	P	12.0	262	96	53	Liver	Locally advanced	Various	59.6	CT scan/SMI	113	86	27	N	F
Chen WS et al., 2020 [76]	Y	P	0.0	360	214	146	Colorectal	Various	Surgery	72.0	AWGS 1/SMI	133	76	57	N	G
Fraisse G et al., 2020 [77]	N	RP	64.8	146	126	20	Urothelial	Various	Various	NA	CT scan/SMI	67	59	8	N	P
Hirsch L et al.,2020 [78]	N	P	18.0	92	58	34	Various	Metastatic	Immunotherapy	64.6	CT scan/SMI	45	NA	NA	N	G
Huang CH et al., 2020 [79]	Y	RP	84.0	107	101	6	Esophageal	Various	Various	54.1	CT scan/SMI	65	63	2	Y	P
Lanza E et al., 2020 [80]	N	RP	60.0	142	110	32	Liver	Various	Intra-arterial infusion for hepatocellular carcinoma	73.0	CT scan/SMI	121	97	24	Y	G
Tsukagoshi M et al., 2020 [81]	Y	RP	36.0	30	23	7	Lung NSC	Various	Immunotherapy	67.0	CT scan/PMI	13	10	3	N	G
Ueno A et al., 2020 [82]	Y	RP	0.0	82	0	82	Breast	Various	Chemotherapy	54.0	CT scan/SMI	10	NA	10	Y	F
Pielkenrood BJ et al., 2020 [83]	N	P	19.2	310	194	116	Various	Metastatic	Radiotherapy	67.0	CT scan/SMI	119	NA	NA	N	G
Wang PY et al., 2020 [84]	Y	P	0.2	212	145	67	Esophageal	Various	Surgery	64.9	AWGS 1/ASM	55	37	18	Y	G
Martini K et al., 2020 [85]	N	RP	1.0	234	69	165	Lung NSC	Various	Surgery	NA	CT scan/NA	78	23	55	N	F
Berardi G et al., 2020 [86]	N	P	3.0	234	158	76	Various	Various	Surgery	66.5	EWGOS 2/NA	68	31	37	Y	G
den Boer RB et al., 2020 [87]	N	P	3.0	199	158	41	Gastric	Various	Various	66.1	CT scan/SMI	84	67	17	N	F
Xu LB et al., 2020 [88]	Y	P	48.0	749	499	250	Gastric	Various	Surgery	NA	AWGS 2/SMI	134	91	43	Y	G
Yu J II et al., 2020 [89]	Y	RCT	192.0	458	282	176	Gastric	Various	Various	NA	CT scan/SMI	75	74	1	Y	G
Mishra A et al., 2020 [90]	N	P	0.0	296	161	135	Leukemia	Metastatic	Chemotherapy	52.4	CT scan/SMI	132	75	57	N	G
Choi K et al., 2020 [91]	Y	P	72.0	238	193	45	Liver	Various	Various	59.0	CT scan/PMI	135	130	5	Y	G
Benadon B et al., 2020 [92]	N	P	60.0	104	72	32	Esophageal	Locally advanced	Various	63.0	CT scan/SMI	84	NA	NA	Y	G
Mallet R et al., 2020 [93]	N	P	120.0	97	81	16	Esophageal	Various	Various	63.6	CT scan/SMI	54	49	5	Y	G
Ryu Y et al., 2020 [94]	Y	P	60.0	548	326	222	Pancreas	Various	Various	62.5	CT scan/SMI	252	186	66	Y	G
Giani A et al., 2020 [95]	N	P	0.0	173	111	62	Colorectal	Local	Surgery	70.0	CT scan/NA	43	NA	NA	Y	F
van Rijn-Dekker MI et al., 2020 [96]	N	P	60.0	750	555	195	Head and neck	Various	Various	NA	CT scan/NA	189	143	46	Y	G
Srpcic M et al., 2020 [97]	N	P	120.0	139	117	22	Esophageal	Various	Surgery	63.9	CT scan/SMI	23	20	3	Y	G
Roch B et al., 2020 [98]	N	RP	30.0	142	93	49	Lung NSC	Metastatic	Immunotherapy	63.5	CT scan/SMI	92	NA	NA	Y	G
Agalar C et al., 2020 [99]	N	P	36.0	65	23	42	Colorectal	Metastatic	Various	56.0	CT scan/SMI	20	6	14	N	F
Shinohara S et al., 2020 [100]	Y	RP	96.0	391	275	116	Lung NSC	Various	Surgery	69.3	CT scan/PMI	198	160	38	Y	G
Salman MA et al., 2020 [101]	N	P	12.0	52	38	14	Liver	Local	Surgery	53.9	CT scan/SMI	27	18	9	N	G
Stangl-Kremser J et al., 2020 [102]	N	RCT	60.0	186	186	0	Prostate	Metastatic	Chemotherapy	68.8	CT scan/SMI	154	154	NA	Y	G
Zhuang CL et al., 2020 [103]	Y	RP	36.0	883	619	264	Gastric	Various	Surgery	65.0	EWGOS 1/EWGOS 2/SMI	150	103	47	Y	G
Hendrickson NR et al., 2020 [104]	N	RP	12.0	145	83	62	Sarcoma	Various	Surgery	NA	CT scan/PMI	38	21	17	Y	G
Yumioka T et al., 2020 [105]	Y	RP	0.0	80	55	25	Various	Various	Chemotherapy	71.6	CT scan/TPA	39	NA	NA	N	F
Oflazoglu U et al., 2020 [106]	N	P	0.0	461	203	258	Various	Various	Not specified	58.2	EWGOS 1/ASM	77	59	18	Y	G
Lee EC et al., 2020 [107]	Y	P	60.0	158	73	85	Urothelial	Various	Surgery	64.0	CT scan/SMI	88	58	30	Y	F
Martin L et al., 2020 [108]	N	P	19.6	1157	744	413	Various	Various	Various	63.6	CT scan/SMI	173	NA	NA	Y	F
Couderc AL et al., 2020 [109]	N	P	0.0	31	31	0	Prostate	Various	Various	80.4	EWGOS 2/ASM	8	8	NA	N	F
He WZ et al., 2020 [110]	Y	P	144.0	1767	1382	385	Head and neck	Various	Various	NA	CT scan/SMI	683	573	110	N	G
Chen XY et al., 2019 [111]	Y	P	0.0	313	229	84	Gastric	Various	Surgery	62.0	AWGS 1/SMI	37	23	14	Y	G
Dijksterhuis WPM et al., 2019 [112]	N	P	0.0	88	66	22	Various	Metastatic	Chemotherapy	63.0	CT scan/SMI	43	29	14	N	F
Dolan RD et al., 2019 [113]	N	P	109.2	650	354	296	Colorectal	Various	Surgery	NA	CT scan/SMI	283	150	133	N	G
de Paula N et al., 2019 [114]	N	RP	13.0	232	0	232	Uterus	Various	Various	64.3	CT scan/SMI	60	0	60	Y	F
Griffin OM et al., 2019 [115]	N	P	48.0	78	37	41	Pancreas	Various	Chemotherapy	64.2	CT scan/SMI	39	NA	NA	N	F
Hopkins JJ et al., 2019 [116]	N	RP	123.0	968	589	379	Colorectal	Various	Surgery	65.8	CT scan/SMI	488	262	226	Y	G
Jung A et al., 2019 [117]	Y	P	70.5	258	223	35	Head and neck	Various	Various	64.0	CT scan/SMI	17	NA	NA	Y	G
Huillard O et al., 2019 [118]	N	RCT	0.0	180	NA	NA	Thyroid	Metastatic	Targeted therapy	63.0	CT scan/SMI	89	NA	NA	Y	G
Kitano Y et al., 2019 [119]	Y	RP	94.1	110	75	35	Cholangiocarcinoma	Locally advanced	Surgery	71.0	CT scan/SMI	31	17	14	N	G
Kurk S et al., 2019 [120]	N	RCT	57.0	182	115	67	Colorectal	Metastatic	Chemotherapy	64.0	CT scan/NA	99	63	36	Y	G
Lin J et al., 2019 [121]	Y	P	0.0	594	448	146	Gastric	Locally advanced	Surgery	64.3	CT scan/SMI	195	NA	NA	N	G
Matsunaga T et al., 2019 [122]	Y	RP	54.0	163	128	35	Esophageal	Various	Various	64.7	BIA/NA	82	64	18	Y	G
Tamura T et al., 2019 [123]	Y	RP	1.0	153	101	52	Gastric	Various	Surgery	NA	BIA/NA	24	17	7	Y	G
Vashi PG et al., 2019 [124]	N	RP	70.0	112	63	49	Colorectal	Various	Various	56.3	CT scan/SMI	46	26	20	N	G
Yamamoto K et al., 2019 [125]	Y	RP	60.0	90	61	29	Gastric	Various	Surgery	NA	EWGOS 1/ASM	19	17	2	Y	G
Yang J et al., 2019 [126]	Y	RP	1.0	417	251	166	Colorectal	Various	Surgery	57.9	CT scan/SMI	61	42	19	Y	G
Okabe H et al., 2019 [127]	Y	RP	0.0	269	167	102	Colorectal	Various	Surgery	74.0	CT scan/SMI	159	81	78	Y	G
Otten L et al., 2019 [128]	N	P	12.0	439	248	191	Various	Various	Various	69.6	EWGOS 1/ASM	119	82	37	Y	G
Panje CM et al., 2019 [129]	N	RCT	84.0	61	57	4	Esophageal	Locally advanced	Various	61.0	CT scan/SMI	31	NA	NA	N	G
Sasaki S et al., 2019 [130]	Y	RCT	6.0	219	143	76	Colorectal	Various	Various	64.0	CT scan/SMI	135	109	26	Y	G
Shi B et al., 2019 [131]	Y	RP	0.0	279	205	74	Gastric	Various	Surgery	56.2	CT scan/SMI	125	106	19	N	G
da Silva JR et al., 2019 [132]	N	P	13.0	334	151	183	Various	Various	Palliative	63.0	CT scan/ASM	219	NA	NA	Y	G
Charette N et al., 2019 [133]	N	RCT	30.0	217	123	94	Colorectal	Locally advanced	Chemotherapy	63.0	CT scan/SMI	150	NA	NA	Y	G
Jang M et al., 2019 [134]	Y	RP	0.0	284	163	121	Pancreas	Local	Surgery	62.6	CT scan/SMI	191	NA	NA	Y	G
Kiss N et al., 2019 [135]	N	P	80.0	41	29	12	Lung NSC	Various	Various	65.6	CT scan/SMI	25	NA	NA	Y	G
Kurita Y et al., 2019 [136]	Y	RP	66.0	82	60	22	Pancreas	Metastatic	Chemotherapy	64.0	CT scan/SMI	42	31	11	N	F
Nakamura N et al., 2019 [137]	Y	RP	146.5	90	51	39	Lymphoma	Metastatic	Chemotherapy	59.0	CT scan/SMI	39	25	14	N	F
Ma BW et al., 2019 [138]	Y	P	1.0	545	418	127	Gastric	Locally advanced	Surgery	62.6	EWGOS 1/SMI	40	25	15	Y	G
Wang P et al., 2019 [139]	Y	P	12.0	44	26	18	Esophageal	Various	Surgery	65.7	BIA/NA	18	NA	NA	N	G
Soma D et al., 2019 [140]	Y	P	0.0	102	89	13	Esophageal	Various	Various	67.3	CT scan/SMI	45	34	11	N	G
Zhang S et al., 2019 [141]	Y	RP	43.2	6447	4317	2130	Various	Various	Surgery	NA	CT scan/NA	1638	1109	529	Y	F
Ataseven B et al., 2018 [142]	N	RP	0.0	323	0	323	Ovary	Various	Surgery	60.0	CT scan/SMI	152	NA	152	Y	G
Banaste N et al., 2018 [143]	N	RP	81.6	214	105	109	Colorectal	Metastatic	Various	59.5	CT scan/SMI	90	NA	NA	N	G
Chambard LC et al., 2018 [144]	N	P	50.0	64	48	16	Lung NSC	Metastatic	Various	65.1	DXA/ASM	16	NA	NA	N	G
Chen WZ et al., 2018 [145]	Y	P	0.0	376	228	148	Colorectal	Various	Surgery	64.3	AWGS 1/SMI	92	44	48	Y	G
Kawamura T et al., 2018 [146]	Y	RP	66.5	951	660	291	Gastric	Various	Surgery	74.2	AWGS 1/AMA	111	69	42	Y	G
Ní Bhuachalla EB et al., 2018 [147]	N	P	26.1	725	433	292	Various	Various	Chemotherapy	64.3	CT scan/NA	274	144	130	N	G
Kim YR et al., 2018 [148]	Y	RP	80.0	92	92	0	Liver	Metastatic	Surgery	54.0	CT scan/NA	72	72	0	Y	G
Lee JS et al., 2018 [149]	Y	RP	31.9	140	106	34	Gastric	Various	Chemotherapy	67.0	CT scan/SMI	67	66	1	N	F
Mayr R et al., 2018 [150]	N	RP	3.0	327	262	65	Urothelial	Various	Surgery	70.0	CT scan/SMI	108	81	27	N	G
Mao CC et al., 2018 [151]	Y	P	1.2	682	513	169	Gastric	Various	Surgery	64.5	AWGS 1/SMI	132	90	42	Y	F
Motoori M et al., 2018 [152]	Y	RP	0.0	83	66	17	Esophageal	Various	Various	65.0	BIA/ASM	28	55	NA	N	P
McSorley ST et al., 2018 [153]	N	P	96.0	322	174	148	Colorectal	Various	Surgery	NA	CT scan/SMI	158	NA	NA	Y	F
van der Kroft G et al., 2018 [154]	N	P	1.0	63	39	24	Colorectal	Various	Surgery	69.0	CT scan/SMI	33	20	13	N	F
van Vugt JLA et al., 2018 [155]	N	P	1.0	816	440	376	Colorectal	Various	Surgery	NA	CT scan/SMI	411	NA	NA	Y	G
Williams GR et al., 2018 [156]	N	P	0.1	25	12	13	Colorectal	Various	Chemotherapy	59.0	CT scan/SMI	12	NA	NA	N	F
Zhang WT et al., 2018 [157]	Y	RP	1.0	636	478	158	Gastric	Various	Surgery	NA	AWGS 1/SMI	86	64	22	Y	G
Zhang Y et al., 2018 [158]	Y	RP	0.2	156	115	41	Gastric	Various	Surgery	59.1	CT scan/SMI	24	17	7	Y	G
Okugawa Y et al., 2018 [159]	Y	P	60.0	167	99	68	Colorectal	Various	Various	67.0	CT scan/PMI	55	20	35	N	F
Rier HN et al., 2018 [160]	N	P	0.0	131	73	58	Various	Various	Various	72.0	EWGOS 1/SMI	34	18	16	Y	F
Sato S et al., 2018 [161]	Y	RP	36.0	48	32	16	Esophageal	Locally advanced	Various	65.5	CT scan/SMI	34	23	11	Y	F
Stretch C et al., 2018 [162]	N	RP	120.0	123	61	52	Pancreas	Various	Surgery	68.5	CT scan/SMI	50	29	21	N	F
Sugimoto M et al., 2018 [163]	N	RP	60.0	323	176	147	Pancreas	Various	Various	65.0	CT scan/SMI	200	NA	NA	Y	G
Sui K et al., 2018 [164]	Y	P	60.0	354	203	151	Pancreas	Various	Surgery	70.0	CT scan/SMI	87	51	36	Y	G
Limpawattana P et al., 2018 [165]	Y	P	30.0	75	58	17	Bile ducts	Various	Various	57.0	AWGS 1/ASM	40	40	6	Y	F
Caan BJ et al., 2018 [166]	N	RP	120.0	3241	0	3241	Breast	Various	Various	54.1	CT scan/SMI	1086	0	1086	N	G
Ha Y et al., 2018 [167]	Y	RP	96.0	178	141	37	Liver	Various	Various	NA	CT scan/SMI	62	43	19	N	G
Nakashima Y et al., 2018 [168]	Y	RP	60.0	341	289	52	Esophageal	Various	Surgery	NA	CT scan/SMI	171	NA	NA	Y	G
Makiura D et al., 2018 [169]	Y	P	60.0	98	83	15	Esophageal	Various	Surgery	67.0	AWGS 1/ASM	31	24	7	N	F
Mason RJ et al., 2018 [170]	N	RP	84.0	698	698	0	Prostate	Various	Surgery	61.8	CT scan/SMI	388	388	0	Y	G
Begini P et al., 2017 [171]	N	RP	100.0	92	65	27	Liver	Various	Various	71.6	CT scan/SMI	37	20	17	N	G
Black D et al., 2017 [172]	N	RP	61.0	447	256	191	Various	Various	Various	NA	CT scan/SMI	104	NA	NA	Y	G
Daly LE et al., 2017 [173]	N	RP	0.0	84	52	32	Melanoma	Metastatic	Immunotherapy	54.0	CT scan/SMI	20	10	10	Y	G
Endo T et al., 2017 [174]	Y	P	0.0	121	81	40	Various	Various	Surgery	70.3	BIA/ASM	29	NA	NA	Y	F
Härter J et al., 2017 [175]	N	P	5.0	60	34	26	Various	Various	Surgery	NA	BIA/ASM	11	NA	NA	Y	P
Heidelberger V et al., 2017 [176]	N	RP	17.0	68	36	32	Melanoma	Various	Immunotherapy	65.0	CT scan/SMI	34	NA	NA	N	F
Huang DD et al., 2017 [177]	Y	P	1.0	470	364	106	Gastric	Various	Surgery	65.0	AWGS 1/SMI	79	59	20	Y	F
Imai K et al., 2017 [178]	Y	RP	0.0	351	242	109	Liver	Various	Various	70.4	CT scan/SMI	33	30	3	Y	G
Paireder M et al., 2017 [235]	N	RP	99.4	130	106	24	Esophageal	Various	Various	61.4	CT scan/SMI	80	68	12	Y	G
Lou N et al., 2017 [179]	Y	P	1.0	206	161	45	Gastric	Various	Surgery	64.0	AWGS 1/SMI	14	9	5	Y	G
Cushen SJ et al., 2017 [180]	N	RP	0.0	55	43	12	Kidney	Metastatic	Targeted therapy	66.0	CT scan/SMI	18	18	0	N	G
Cespedes Feliciano EMC et al., 2017 [181]	N	P	120.0	2470	1251	1219	Colorectal	Various	Surgery	63.0	CT scan/SMI	1133	NA	NA	Y	G
Elliott JA et al., 2017 [182]	N	P	60.0	207	165	42	Esophageal	Various	Surgery	61.6	CT scan/SMI	49	45	4	Y	F
Wendrich AW et al., 2017 [183]	N	RP	90.0	112	72	40	Head and neck	Various	Chemotherapy	54.5	CT scan/SMI	61	23	38	Y	G
Bronger H et al., 2017 [184]	N	RP	60.0	128	0	128	Ovary	Various	Various	65.0	CT scan/SMI	16	0	16	Y	G
Ishihara H et al., 2017 [185]	Y	RP	58.0	137	89	48	Urothelial	Locally advanced	Surgery	72.8	CT scan/SMI	90	48	42	Y	F
Miyata H et al., 2017 [186]	Y	P	0.0	94	76	18	Esophageal	Various	Various	64.2	BIA/NA	44	NA	NA	N	G
Zhou CJ et al., 2017 [187]	Y	P	1.0	240	190	50	Gastric	Various	Surgery	73.0	AWGS 1/SMI	69	52	17	Y	F
Chemama S et al., 2016 [188]	N	RP	0.0	97	37	60	Colorectal	Metastatic	Various	53.0	CT scan/SMI	39	6	33	N	G
Grotenhuis BA et al., 2016 [189]	N	RCT	104.0	120	88	32	Esophageal	Locally advanced	Various	62.0	CT scan/SMI	54	42	12	N	G
Nishigori T et al., 2016 [190]	Y	RP	0.0	199	164	35	Esophageal	Various	Surgery	65.0	CT scan/SMI	149	133	16	Y	G
Okumura S et al., 2016 [191]	Y	RP	60.0	207	111	96	Bile ducts	Various	Surgery	68.0	CT scan/SMI	71	36	35	N	G
Pecorelli N et al., 2016 [192]	N	RP	2.0	202	108	94	Pancreas	Various	Surgery	66.8	CT scan/SMI	132	79	53	Y	F
Park I et al., 2016 [193]	Y	P	44.3	88	59	29	Various	Metastatic	Chemotherapy	65.0	CT scan/ASM	76	57	19	Y	F
Suzuki Y et al., 2016 [194]	Y	RP	100.0	90	52	38	Lung NSC	Local	Surgery	68.7	CT scan/SMI	38	16	22	N	F
Takeoka Y et a. 2016 [195]	Y	RP	60.0	56	19	37	Myeloma	Metastatic	Chemotherapy	71.0	CT scan/SMI	37	8	29	Y	F
Fukushima H et al., 2016 [196]	Y	RP	96.0	81	53	28	Urothelial	Various	Surgery	71.0	CT scan/SMI	47	28	19	Y	G
Go SI et al., 2016 [197]	Y	RP	132.0	187	112	75	Lymphoma	Metastatic	Chemotherapy	NA	CT scan/SMI	46	28	18	Y	G
Kumar A et al., 2016 [198]	N	P	60.0	296	0	296	Ovary	Metastatic	Chemotherapy	64.6	CT scan/SMI	132	0	132	N	G
Pędziwiatr M et al., 2016 [199]	N	P	1.0	124	73	51	Colorectal	Various	Surgery	65.9	CT scan/SMI	34	12	22	Y	G
Rollins KE et al., 2016 [200]	N	RP	66.0	228	124	104	Pancreas	Various	Chemotherapy	NA	CT scan/SMI	138	NA	NA	Y	G
Yabusaki N et al., 2016 [201]	Y	RP	60.0	195	157	38	Liver	Local	Surgery	66.0	CT scan/SMI	89	57	32	N	F
Buettner S et al., 2016 [202]	N	P	12.0	1326	730	596	Various	Various	Surgery	62.5	CT scan/TPA	398	219	179	Y	G
Amini N et al., 2015 [203]	N	RP	60.0	763	418	345	Pancreas	Various	Surgery	67.0	CT scan/TPA	192	NA	NA	Y	G
Anandavadivelan P et al., 2015 [204]	N	RCT	0.0	72	61	11	Esophageal	Various	Chemotherapy	67.0	CT scan/SMI	31	NA	NA	N	G
Fukuda Y et al., 2015 [205]	Y	P	0.0	99	66	33	Gastric	Various	Surgery	NA	AWGS 1/ASM	21	19	2	Y	G
Huang DD et al., 2015 [206]	Y	P	1.0	142	88	54	Colorectal	Various	Surgery	62.0	AWGS 1/SMI	17	11	6	Y	G
Ida S et al., 2015 [207]	Y	P	0.0	138	121	17	Esophageal	Various	Surgery	NA	BIA/NA	61	47	14	N	G
Kim EY et al., 2015 [208]	Y	RP	38.0	149	127	22	Lung SC	Various	Various	68.6	CT scan/SMI	118	110	8	Y	G
Levolger S et al., 2015 [209]	N	P	36.0	90	63	27	Liver	Various	Various	62.0	CT scan/SMI	52	39	13	Y	G
Reisinger KW et al., 2015 [210]	N	RP	1.0	310	155	155	Esophageal	Various	Surgery	69.0	CT scan/SMI	148	90	58	Y	G
Tamandl D et al., 2015 [211]	N	RP	60.0	200	151	49	Esophageal	Various	Surgery	63.9	CT scan/SMI	130	107	23	Y	G
Tegels JJ et al., 2015 [212]	N	RP	6.0	149	NA	NA	Gastric	Various	Surgery	69.8	EWGOS 1/SMI	86	NA	NA	Y	G
Voron T et al., 2015 [213]	N	RP	70.0	109	92	17	Liver	Local	Surgery	61.7	CT scan/SMI	59	53	6	Y	G
Lodewic TM et al., 2015 [214]	N	P	60.0	171	104	67	Colorectal	Metastatic	Surgery	64.0	CT scan/SMI	80	45	35	N	G
Tan BH et al., 2015 [215]	N	RP	83.3	89	67	22	Various	Various	Chemotherapy	65.8	CT scan/SMI	44	34	10	N	G
Wang SL et al., 2015 [216]	Y	P	1.0	255	190	65	Gastric	Various	Surgery	65.1	AWGS 1/SMI	32	26	6	Y	G
van Vugt JL et al., 2015 [217]	N	RP	1.0	206	100	106	Colorectal	Metastatic	Surgery	NA	CT scan/SMI	90	46	44	N	G
Gonzalez MC et al., 2014 [218]	N	P	36.0	175	60	115	Various	Various	Chemotherapy	56.9	BIA/ASM	22	NA	NA	Y	G
Barret M et al., 2014 [219]	N	P	2.0	51	38	13	Colorectal	Metastatic	Chemotherapy	65.0	CT scan/SMI	36	31	5	Y	G
Harimoto N et al., 2013 [220]	Y	RP	60.0	186	145	41	Liver	Various	Surgery	NA	CT scan/SMI	75	50	25	N	G
Huillard O et al., 2013 [221]	N	RP	52.0	61	38	59	Kidney	Metastatic	Targeted therapy	60.0	CT scan/SMI	32	24	8	N	G
Veasey-Rodrigues H et al., 2013 [222]	N	P	2.0	16	5	11	Various	Metastatic	Targeted therapy	60.0	CT scan/SMI	7	NA	NA	Y	F
Veasey-Rodrigues H et al., 2013 [223]	N	RCT	3.0	306	159	147	Various	Metastatic	Various	56.0	CT scan/SMI	144	93	51	Y	G
Meza-Junco J et al., 2013 [224]	N	RP	24.0	116	98	18	Liver	Various	Various	58.0	CT scan/SMI	35	30	5	N	F
Lieffers JR et al., 2012 [225]	N	RP	1.0	234	135	99	Colorectal	Various	Surgery	63.0	CT scan/SMI	91	57	34	N	G
Mir O et al., 2012 [226]	N	RP	16.0	18	15	3	Liver	Metastatic	Chemotherapy	64.0	CT scan/SMI	9	NA	NA	N	F
Parsons HA et al., 2012 [227]	N	RCT	26.6	104	65	39	Various	Metastatic	Various	NA	CT scan/SMI	53	36	17	N	G
Parsons HA et al., 2012 [228]	N	RP	26.6	48	19	29	Various	Metastatic	Intra-arterial infusion for hepatocellular carcinoma	56.0	CT scan/SMI	21	10	11	N	F
van Vledder MG et al., 2012 [229]	N	RP	97.0	196	120	76	Colorectal	Metastatic	Surgery	64.5	CT scan/SMI	38	11	27	Y	G
Dalal S et al., 2012 [230]	N	RCT	90.0	41	18	23	Pancreas	Locally advanced	Various	58.9	CT scan/SMI	26	NA	NA	Y	F
Antoun S et al., 2010 [231]	N	RCT	6.0	80	60	20	Kidney	Metastatic	Targeted therapy	59.8	CT scan/SMI	42	20	13	Y	G
Tan BH et al., 2009 [232]	N	P	42.0	111	52	59	Pancreas	Various	Palliative	64.4	CT scan/SMI	62	33	27	Y	G
Prado CM et al., 2009 [233]	N	P	19.2	55	NA	55	Breast	Metastatic	Targeted therapy	54.8	CT scan/SMI	14	NA	14	N	G
Prado CM et al., 2008 [234]	N	P	39.6	250	136	114	Various	Various	Not specified	63.9	CT scan/SMI	38	28	10	Y	F

Y = yes; N = no; RCT = randomized control trial; P = prospective observational study; RP = retrospective observational study with consecutive inclusion; M = male; F = female; NSC = non-small cell; SC = small cell; NA = not available; BIA = bioelectrical impedance analysis; DXA = dual energy X-ray absorptiometry; AMA = arm muscle area; ASM = appendicular skeletal muscle mass; SMI = skeletal muscle index; PMI = psoas muscle index; TPA = total psoas area; AWGS = Asian Working Group on Sarcopenia; EWGOS = European Working Group On Sarcopenia; NOS score: G = good; F = fair; P = poor.

**Table 2:** Prevalence of sarcopenia among cancer patients.

Study Groups	Patients		Prevalence % [95% CI]	*p* Value for Subgroup Differences	Heterogeneity	
	N	(%)			I^2^	*p*
**Overall**				0.11		
All studies	65,936	(100)	38.0 [36.0–41.0]		97%	<0.01
Excluding studies over the 95% CI (funnel plot)	18,935	(29)	40.5 [39.0–42.0]		66%	<0.01
**Quality of study (NOS scale)**				0.75		
Good	47,028	(71)	38.0 [34.0–41.0]		97%	<0.01
Fair	18,287	(28)	40.0 [35.0–44.0]		96%	<0.01
Poor	621	(1)	40.5 [28.5–54.0]		86.5%	<0.01
**Year of publication**				0.80		
2008–2012	1343	(2)	40.0 [30.0–50.0]		92%	<0.01
2013–2017	13,411	(20)	40.0 [34.0–46.0]		96%	<0.01
2018–2022	51,182	(78)	38.0 [35.0–41.0]		97%	<0.01
**N° of patients included**				**<0.01**		
<100	4364	(7)	45.0 [41.0–50.0]		84%	<0.01
<100–199	9606	(14.5)	41.0 [36.0–47.0]		95%	<0.01
<200–399	16,023	(24)	36.0 [31.0–42.0]		97%	<0.01
≥400	35,943	(54.5)	27.0 [22.0–32.0]		99%	<0.01
**World region**				0.26		
Asia	33,453	(51)	37.0 [32.0–41.0]		97.5%	<0.01
Not Asia	32,483	(49)	40.0 [37.0–43.0]		95%	<0.01
**Mean or median age (y) at inclusion (n = 47,986)**				0.21		
<65	30,691	(64)	38.0 [34.0–42.0]		96%	<0.01
≥65	17,295	(36)	42.0 [37.0–46.0]		96%	<0.01
**Sex (n = 46,265)**				0.22		
Women	15,841	(34)	34.0 [30.0–38.0]		91%	<0.01
Men	30,424	(66)	37.0 [34.0–41.0]		96%	<0.01
**BMI (n = 8627)**				**<0.01**		
≥30 kg/m^2^	2628	(30.5)	19.0 [13.0–27.0]		89.5%	<0.01
<30 kg/m^2^	5999	(69.5)	39.0 [31.0–47.0]		96%	<0.01
**Cancer site**				**<0.01**		
Gastric	13,513	(20.5)	24.0 [19.0–29.5]		97.5%	<0.01
Breast	3517	(5)	25.0 [17.5–35.0]		82%	<0.01
Sarcoma	254	(0.4)	25.0 [20.0–30.5]		0%	0.55
Uterus	232	(0.3)	26.0 [21.0–32.0]		-	-
Head and neck	3724	(6)	31.0 [21.0–43.0]		95%	<0.01
Ovarian	747	(1)	33.0 [16.0–55.0]		95%	<0.01
Lymphoma	1130	(2)	35.0 [29.0–41.5]		75%	<0.01
Various	14,600	(22)	35.0 [29.0–41.0]		96%	<0.01
Cholangiocarcinoma	231	(0.3)	36.0 [26.0–47.0]		83%	<0.01
Melanoma	152	(0.2)	36.0 [20.0–56.0]		91%	<0.01
Leukemia	474	(0.7)	36.0 [25.0–49.0]		93%	<0.01
Colorectal	11,419	(17)	38.0 [33.0–44.0]		95%	<0.01
Anal	106	(0.2)	39.0 [30.0–48.0]		-	-
Bile ducts	282	(0.4)	42.5 [30.0–56.0]		88%	<0.01
Non-small cell lung	2914	(4)	43.0 [34.0–51.5]		95%	<0.01
Liver	2391	(4)	44.0 [33.0–55.5]		95%	<0.01
Myeloma	152	(0.2)	44.0 [18.0–74.0]		95%	<0.01
Thyroids	180	(0.3)	49.5 [42.0–57.0]		-	-
Pancreatic	3813	(6)	49.5 [41.5–57.5]		96%	<0.01
Kidney	356	(0.5)	50.0 [43.0–57.0]		53%	0.07
Esophageal	3474	(5)	50.0 [43.0–57.0]		92%	<0.01
Urothelial	1163	(2)	52.0 [39.5–64.0]		94%	<0.01
Prostatic	985	(1.5)	60.0 [38.0–79.0]		95%	<0.01
Small cell lung	149	(0.2)	79.0 [72.0–85.0]		-	-
**Cancer extension**				**<0.01**		
Various	54,269	(82)	35.0 [32.0–38.0]		97%	<0.01
Local	2783	(4)	39.0 [30.0–50.0]		97%	<0.01
Locally advanced	3186	(5)	48.0 [37.0–59.0]		96%	<0.01
Metastatic	5698	(9)	46.0 [40.0–51.0]		92%	<0.01
**Treatment modalities**				**<0.01**		
Not specified	918	(1)	21.0 [12.5–33.0]		91%	<0.01
Surgery	40,486	(61)	33.0 [30.0–37.0]		97%	<0.01
Targeted therapy	634	(1)	41.0 [32.0–50.0]		81%	<0.01
Various	17,641	(27)	41.0 [36.0–45.0]		96%	<0.01
Immune therapy	909	(1)	46.0 [38.0–54.5]		80%	<0.01
Radiotherapy	544	(0.8)	46.0 [28.0–66.0]		93%	<0.01
Chemotherapy	4169	(6)	48.0 [41.0–56.0]		93%	<0.01
Exclusive supportive care	445	(0.7)	62.0 [55.0–69.0]		70%	<0.01
Intra-arterial infusion for hepatocellular carcinoma	190	(0.3)	68.0 [35.0–90.0]		96.5%	<0.01
**Definition of sarcopenia**				**<0.01**		
Consensus algorithm-based						
Overall	11,013	(17)	22.0 [19.0–26.0]		93%	<0.01
AWGS	6996		20.5 [16.0–25.0]		93%	<0.01
EWGOS 2	2462		20.5 [15.5–27.0]		88%	<0.01
EWGOS 1	3086		25.0 [17.0–35.0]		96%	<0.01
Muscle mass quantity only						
Overall	54,923	(83)	42.0 [39.0–45.0]		96%	<0.01
DXA	64		25.0 [16.0–37.0]		-	-
BIA	1306		30.0 [23.0–38.0]		90%	<0.01
CT scan	53,553		43.0 [40.0–46.0]		96.5%	<0.01
**Muscle mass indices (n = 55,304)**				**<0.01**		
AMA (cm^2^)	951	(2)	12.0 [10.0–14.0]		-	-
ASM (kg/m^2^)	3261	(6)	31.0 [24.0–39.0]		95%	<0.01
TPA (cm^2^/m^2^)	2394	(4)	36.0 [27.0–46.0]		93%	<0.01
PMI (cm^2^/m^2^)	2967	(5)	36.5 [28.0–46.0]		96.5%	<0.01
SMI (cm^2^/m^2^)	45,731	(83)	40.0 [37.0–43.0]		97%	<0.01
**Median cut-off values of CT scan-based SMI for women (n = 14,216)**				**<0.01**		
<38.5 (cm^2^/m^2^)	4609	(32)	25.0 [21.0–29.0]		87%	<0.01
≥38.5 (cm^2^/m^2^)	9607	(68)	47.0 [40.0–54.0]		92%	<0.01
**Median cut-off values of CT scan-based SMI for men (n = 20,514)**				**<0.01**		
<47.3 (cm^2^/m^2^)	11,584	(56)	28.0 [24.0–32.0]		95%	<0.01
≥47.3 (cm^2^/m^2^)	8930	(44)	52.0 [46.0–58.0]		95%	<0.01

Bold = grouping data, and significant *p* value at the threshold of 5%; BIA = bioelectrical impedance analysis; DXA = dual energy X-ray sbsorptiometry; AWGS = Asian Working Group on Sarcopenia; EWGOS = European Working Group On Sarcopenia; AMA = arm muscle area; ASM = appendicular skeletal muscle mass; SMI = skeletal muscle index; PMI = psoas muscle index; TPA = total psoas area.

**Table 3:** Predictive value of pre-therapeutic sarcopenia on overall survival (OS) among cancer patients.

Study Groups	Patients		Relative Risk [95% CI] for OS	*p* Value for Subgroup Differences	Heterogeneity	
	N	(%)			I^2^	*p*
**Overall**				0.37		
All studies	28,995	(100)	1.97 [1.79–2.17]		85%	<0.01
Excluding studies over the 95% CI (funnel plot)	7191	(25)	1.68 [1.55–1.80]		77%	<0.01
**Quality of study (NOS)**				0.65		
Good	22,939	(79)	1.94 [1.73–2.16]		75%	<0.01
Fair	5803	(20)	2.10 [1.71–2.58]		90.5%	<0.01
Poor	253	(1)	1.40 [0.47–4.20]		77%	0.04
**Year of publication**				0.93		
2008–2012	598	(2)	1.85 [1.29–2.65]		54%	0.09
2013–2017	5977	(21)	1.97 [1.53–2.52]		93%	<0.01
2018–2022	22,420	(77)	1.99 [1.78–2.22]		75%	<0.01
**N° of patients included**				**0.01**		
<100	1718	(6)	2.24 [1.71–2.92]		87%	<0.01
100–199	5150	(18)	2.17 [1.86–2.53]		61%	<0.01
200–399	8417	(29)	1.90 [1.57–2.30]		81%	<0.01
≥400	13,710	(47)	1.57 [1.35–1.82]		71%	<0.01
**World region**				**<0.01**		
Asia	10,964	(38)	2.37 [2.07–2.71]		84%	<0.01
Not Asia	18,031	(62)	1.69 [1.48–1.91]		72%	<0.01
**Mean or median age (y) at inclusion (n = 23,630)**				0.09		
<65	14,384	(61)	1.87 [1.62–2.16]		75%	<0.01
≥65	9246	(39)	2.24 [1.93–2.61]		89%	<0.01
**Cancer site**				**<0.01**		
Gastric	5447	(19)	1.88 [1.46–2.44]		74%	<0.01
Breast	0	(0)	-		-	-
Sarcoma	145	(0.5)	3.42 [0.81–14.4]		-	-
Uterus	232	(0.8)	2.23 [1.18–3.92]		-	-
Head and neck	1692	(6)	2.75 [2.00–3.78]		62%	0.03
Ovarian	424	(1.5)	1.64 [0.53–5.06]		82.5%	0.02
Lymphoma	997	(3)	1.55 [0.89–2.70]		73%	0.02
Various	3649	(13)	1.72 [1.19–2.45]		96%	<0.01
Cholangiocarcinoma	231	(0.8)	2.66 [1.85–3.81]		0%	0.69
Melanoma	0	(0)	-		-	-
Leukemia	178	(0.6)	3.12 [1.53–6.35]		-	-
Colorectal	7252	(25)	1.58 [1.28–1.95]		67%	<0.01
Anal	106	(0.4)	4.50 [1.05–19.2]		-	-
Bile ducts	282	(1)	2.71 [1.87–3.92]		0%	0.49
Non-small cell lung	1440	(5)	2.92 [2.01–4.24]		53%	0.04
Liver	1422	(5)	2.56 [1.94–3.39]		43%	0.07
Myeloma	56	(0.2)	1.96 [0.78–5.00]		-	-
Thyroids	0	(0)	-		-	-
Pancreatic	1789	(6)	1.45 [1.13–1.86]		71%	<0.01
Kidney	78	(0.3)	2.63 [1.50–4.61]		-	-
Esophageal	1856	(6)	2.29 [1.77–2.95]		49%	0.03
Urothelial	835	(3)	1.87 [1.20–2.89]		51%	0.08
Prostatic	884	(3)	1.35 [0.89–2.03]		0%	0.52
Small cell lung	0	(0)	-		-	-
**Cancer extension**				0.40		
Various	23,842	(82)	1.86 [1.68–2.07]		72%	<0.01
Local	1404	(5)	2.32 [1.71–3.15]		27%	0.23
Locally advanced	917	(3)	2.42 [1.50–3.92]		77%	<0.01
Metastatic	2832	(10)	2.09 [1.53–2.86]		94%	<0.01
**Treatment modalities**				0.74		
Not specified	386	(1)	2.16 [1.49–3.13]		0%	0.50
Surgery	16,463	(57)	2.09 [1.84–2.37]		63%	<0.01
Targeted therapy	78	(0.3)	2.63 [1.50–4.61]		-	-
Various	7798	(27)	1.85 [1.55–2.20]		77%	<0.01
Immune therapy	618	(2)	2.37 [0.92–6.08]		92%	<0.01
Radiotherapy	516	(2)	2.91 [1.23–6.90]		77%	0.01
Chemotherapy	2549	(9)	1.70 [1.23–2.36]		95%	<0.01
Exclusive supportive care	445	(1.5)	1.62 [1.06–2.47]		64%	0.10
Intra-arterial infusion for hepatocellular carcinoma	142	(0.5)	2.22 [1.01–4.86]		-	-
**Definition of sarcopenia**				0.24		
Muscle mass quantity only						
CT scan	25,656	(88)	1.93 [1.74–2.15]		86%	<0.01
BIA	347	(1)	1.77 [1.00–2.13]		35%	0.22
DXA	64	(0.2)	2.96 [1.40–6.27]		-	-
Consensus algorithm-based	2928	(10)	2.31 [1.97–2.72]		22%	0.25
**Muscle mass indices (n = 27,061)**				**<0.01**		
AMA (cm^2^)	951	(3)	2.26 [1.69–3.03]		-	-
ASM (kg/m^2^)	1274	(5)	2.84 [2.01–4.00]		87%	<0.01
TPA (cm^2^/m^2^)	0	(0)	-		-	-
PMI (cm^2^/m^2^)	1567	(6)	2.76 [2.21–3.43]		0%	0.63
SMI (cm^2^/m^2^)	23,269	(86)	1.85 [1.66–2.07]		75%	<0.01

Bold = grouping data, and significant *p* value at the threshold of 5%; BIA = bioelectrical impedance analysis; DXA = dual energy X-ray absorptiometry; AMA = arm muscle area; ASM = appendicular skeletal muscle mass; SMI = skeletal muscle index; PMI = psoas muscle index; TPA = total psoas area.

**Table 4:** Predictive value of pre-therapeutic sarcopenia on progression-free survival (PFS) among cancer patients.

Study Groups	Patients		Relative Risk [95% CI] for PFS	*p* Value for Subgroup Differences	Heterogeneity	
	N	(%)			I^2^	*p*
**Overall**				0.23		
All studies	6546	(100)	1.76 [1.44–2.16]		85%	<0.01
Excluding studies over the 95% CI (funnel plot)	4008	(61)	1.35 [1.19–1.52]		80%	<0.01
**Quality of study (NOS)**				0.15		
Good	5055	(77)	1.83 [1.51–2.21]		75%	<0.01
Fair	1345	(20.5)	1.68 [0.89–3.15]		94%	<0.01
Poor	146	(2.5)	0.92 [0.48–1.78]		-	-
**Year of publication**				0.93		
2008–2012	251	(4)	1.89 [1.34–2.64]		0%	0.98
2013–2017	693	(10)	1.83 [1.07–3.13]		83%	<0.01
2018–2022	5602	(86)	1.75 [1.37–2.24]		87%	<0.01
**N° of patients included**				0.73		
<100	489	(7.5)	2.22 [1.24–3.97]			<0.01
100–199	1863	(28.5)	1.81 [1.35–2.42]			<0.01
200–399	1922	(29)	1.49 [0.98–2.24]			<0.01
≥400	2272	(35)	1.79 [0.97–3.29]			<0.01
**World region**				**<0.01**		
Asia	2307	(35)	2.38 [1.81–3.13]		80%	<0.01
Not Asia	4239	(65)	1.42 [1.12–1.81]		75.5%	<0.01
**Mean or median age (y) at inclusion (n = 5891)**				0.78		
<65	3186	(54)	1.75 [1.28–2.40]		91%	<0.01
≥65	2705	(46)	1.85 [1.43–2.39]		60%	0.01
**Cancer site**				**<0.01**		
Gastric	726	(11)	1.68 [0.43–6.50]		96%	<0.01
Breast	55	(0.8)	1.90 [1.03–3.50]		-	-
Sarcoma	109	(2)	4.60 [3.53–6.00]		-	-
Uterus	0	(0)	-		-	-
Head and neck	243	(3.5)	2.45 [1.58–3.78]		-	-
Ovarian	128	(2)	2.64 [1.23–5.64]		-	-
Lymphoma	1040	(16)	1.95 [1.19–3.20]		73%	0.01
Various	349	(5)	0.70 [0.54–0.93]		0%	0.92
Cholangiocarcinoma	0	(0)	-		-	-
Melanoma	0	(0)	-		-	-
Leukemia	0	(0)	-		-	-
Colorectal	2512	(38)	1.35 [1.05–1.74]		55%	0.03
Anal	0	(0)	-		-	-
Bile ducts	207	(3)	2.14 [1.46–3.13]		-	-
Non-small cell lung	534	(8)	2.43 [1.90–3.12]		0%	0.47
Liver	0	(0)	-		-	-
Myeloma	0	(0)	-		-	-
Thyroids	0	(0)	-		-	-
Pancreatic	0	(0)	-		-	-
Kidney	78	(1)	3.18 [1.85–5.47]		-	-
Esophageal	163	(2.5)	1.24 [0.71–2.17]		-	-
Urothelial	146	(2)	0.92 [0.47–1.78]		-	-
Prostatic	256	(4)	2.23 [0.69–7.18]		71%	0.06
Small cell lung	0	(0)	-		-	-
**Cancer extension**				0.13		
Various	4469	(68)	1.62 [1.26–2.08]		81%	<0.01
Local	315	(5)	2.32 [1.62–3.32]		-	-
Locally advanced	47	(1)	8.11 [1.61–41.0]		-	-
Metastatic	1715	(26)	1.84 [1.28–2.64]		89%	<0.01
**Treatment modalities**				0.11		
Not specified	0	(0)	-		-	-
Surgery	3296	(50)	1.73 [1.28–2.35]		81%	<0.01
Targeted therapy	242	(4)	3.21 [1.94–5.33]		73%	0.03
Various	2024	(31)	1.45 [1.10–1.91]		62%	<0.01
Immune therapy	480	(7)	2.11 [0.84–5.29]		90%	<0.01
Radiotherapy	0	(0)	-		-	-
Chemotherapy	504	(8)	1.74 [0.83–3.64]		87%	<0.01
Exclusive supportive care	0	(0)	-		-	-
Intra-arterial infusion for hepatocellular carcinoma	0	(0)	-		-	-
**Definition of sarcopenia**				**<0.01**		
Muscle mass quantity only						
CT scan	5688	(87)	1.70 [1.38–2.10]		84%	<0.01
BIA	163	(2.5)	1.24 [0.71–2.17]		-	-
DXA	0	(0)	-		-	-
Consensus algorithm-based	695	(10.5)	3.59 [2.17–5.92]		12%	0.29
**Muscle mass indices (n = 6383)**				**<0.01**		
AMA (cm^2^)	0	(0)	-		-	-
ASM (kg/m^2^)	47	(1)	8.11 [1.61–40.9]		-	-
TPA (cm^2^/m^2^)	0	(0)	-		-	-
PMI (cm^2^/m^2^)	621	(9.5)	3.05 [2.01–4.62]		72%	0.01
SMI (cm^2^/m^2^)	5715	(87)	1.61 [1.30–1.99]		81%	<0.01

Bold = grouping data, and significant *p* value at the threshold of 5%; BIA = bioelectrical impedance analysis; DXA = dual energy X-ray absorptiometry; AMA = arm muscle area; ASM = appendicular skeletal muscle mass; SMI = skeletal muscle index; PMI = psoas muscle index; TPA = total psoas area.

**Table 5:** Predictive value of pre-therapeutic sarcopenia on severe post-operative complications (POC) among cancer patients.

Study Groups	Patients		Relative Risk [95% CI] for POC	*p* Value for Subgroup Differences	Heterogeneity	
	N	(%)			I^2^	*p*
**Overall**				**0.02**		
All studies	17,172	(100)	2.70 [2.33–3.12]		72%	<0.01
Excluding studies over the 95% CI (funnel plot)	3633	(21)	2.22 [1.84–2.68]		64%	<0.01
**Quality of study (NOS)**				0.34		
Good	14,555	(85)	2.75 [2.34–3.24]		75%	<0.01
Fair	2411	(14)	2.67 [1.83–3.91]		60%	<0.01
Poor	206	(1)	1.87 [1.16–3.04]		0%	0.51
**Year of publication**				**0.02**		
2008–2012	0	(0)	-			
2013–2017	6355	(37)	1.39 [1.18–1.63]		48.5%	<0.01
2018–2022	10,817	(63)	1.91 [1.53–2.38]		81%	<0.01
**N° of patients included**				**0.04**		
<100	806	(5)	1.78 [1.09–2.92]		72%	<0.01
100–199	2425	(14)	1.30 [1.08–1.56]		41%	0.04
200–399	5407	(31)	1.95 [1.42–2.68]		85%	<0.01
≥400	8534	(50)	1.87 [1.47–2.39]		58%	<0.01
**World region**				**<0.01**		
Asia	10,092	(59)	2.02 [1.60–2.55]		81%	<0.01
Not Asia	7080	(41)	1.38 [1.20–1.60]		57%	<0.01
**Mean or median age (y) at inclusion (n = 13,209)**				0.39		
<65	6572	(50)	1.91 [1.49–2.44]		64%	<0.01
≥65	6637	(50)	1.64 [1.28–2.10]		78%	<0.01
**Cancer site**				**<0.01**		
Gastric	6856	(40)	3.09 [2.42–3.93]		43%	0.02
Breast	0	(0)	-		-	-
Sarcoma	145	(1)	1.78 [1.22–2.59]		-	-
Uterus	0	(0)	-		-	-
Head and neck	0	(0)	-		-	-
Ovarian	0	(0)	-		-	-
Lymphoma	0	(0)	-		-	-
Various	1895	(11)	3.95 [1.97–7.95]		71%	<0.01
Cholangiocarcinoma	110	(1)	2.44 [2.08–2.87]		0%	0.46
Melanoma	0	(0)	-		-	-
Leukemia	0	(0)	-		-	-
Colorectal	0	(0)	-		-	-
Anal	0	(0)	-		-	-
Bile ducts	0	(0)	-		-	-
Non-small cell lung	808	(5)	3.66 [1.12–11.9]		93%	<0.01
Liver	385	(2)	2.47 [0.90–6.77]		85%	<0.01
Myeloma	0	(0)	-		-	-
Thyroids	0	(0)	-		-	-
Pancreatic	1629	(9)	1.86 [1.26–2.75]		51%	0.07
Kidney	0	(0)	-		-	-
Esophageal	828	(5)	3.17 [1.82–5.52]		82%	<0.01
Urothelial	473	(3)	1.68 [1.33–2.11]		0%	0.47
Prostatic	698	(4)	4.50 [1.76–11.5]		-	-
Small cell lung	0	(0)	-		-	-
**Cancer extension**				0.06		
Various	14,436	(84)	1.76 [1.46–2.11]		74%	<0.01
Local	999	(6)	2.35 [1.14–4.86]		80%	<0.01
Locally advanced	955	(6)	1.35 [0.85–2.14]		57%	0.05
Metastatic	782	(4)	1.30 [1.09–1.54]		0%	0.80
**Treatment modalities**				**0.04**		
Not specified	0	(0)	-		-	-
Surgery	16,325	(95)	1.77 [1.50–2.09]		76%	<0.01
Targeted therapy	0	(0)	-		-	-
Various	847	(5)	1.26 [0.95–1.67]		43%	0.10
Immune therapy	0	(0)	-		-	-
Radiotherapy	0	(0)	-		-	-
Chemotherapy	0	(0)	-		-	-
Exclusive supportive care	0	(0)	-		-	-
Intra-arterial infusion for hepatocellular carcinoma	0	(0)	-		-	-
**Definition of sarcopenia**				**0.03**		
Muscle mass quantity only						
CT scan	11,212	(65)	2.39 [2.01–2.83]		75%	<0.01
BIA	626	(4)	3.16 [1.74–5.76]		65%	0.02
DXA	0	(0)	-		-	-
Consensus algorithms	5334	(31)	3.62 [2.79–4.69]		36%	0.07
**Muscle mass indices (n = 16,413)**				0.06		
AMA (cm^2^)	0	(0)	-		-	-
ASM (kg/m^2^)	834	(5)	3.26 [1.80–5.90]		72%	<0.01
TPA (cm^2^/m^2^)	2089	(13)	1.60 [1.09–2.35]		72%	0.06
PMI (cm^2^/m^2^)	719	(4)	2.41 [0.99–5.91]		93%	<0.01
SMI (cm^2^/m^2^)	12,771	(78)	1.48 [1.27–1.71]		61%	<0.01

Bold = grouping data, and significant *p* value at the threshold of 5%; BIA = bioelectrical impedance analysis; DXA = dual energy X-ray absorptiometry; AMA = arm muscle area; ASM = appendicular skeletal muscle mass; SMI = skeletal muscle index; PMI = psoas muscle index; TPA = total psoas area.

**Table 6:** Predictive value of pre-therapeutic sarcopenia on severe treatment-related toxicities and/or dose-limiting toxicities (TOX) among cancer patients.

Study Groups	Patients		Relative Risk [95% CI] for TOX	*p* Value for Subgroup Differences	Heterogeneity	
	N	(%)			I^2^	*p*
**Overall**				0.49		
All studies	2980	(100)	1.47 [1.17–1.84]		71%	<0.01
Excluding studies over the 95% CI (funnel plot)	760	(25.5)	1.31 [1.11–1.57]		62%	<0.01
**Quality of study (NOS)**				**0.02**		
Good	2356	(79)	1.34 [1.01–1.77]		67%	<0.01
Fair	517	(17)	1.78 [1.43–2.21]		24%	0.25
Poor	107	(4)	2.72 [1.76–4.21]		-	-
**Year of publication**				0.38		
2008–2012	55	(2)	2.56 [1.14–5.78]		-	-
2013–2017	424	(14)	1.56 [0.94–2.60]		70%	<0.01
2018–2022	2501	(84)	1.40 [1.07–1.84]		73%	<0.01
**N° of patients included**				**0.03**		
<100	851	(28.5)	1.39 [1.01–1.91]		67%	<0.01
100–199	702	(23.5)	1.92 [1.26–2.93]		68.5%	<0.01
200–399	219	(7.5)	0.98 [0.78–1.25]		-	-
≥400	1208	(40.5)	1.42 [0.63–3.21]		88%	<0.01
**World region**				0.80		
Asia	1551	(52)	1.43 [1.04–1.98]		76.5%	<0.01
Not Asia	1429	(48)	1.52 [1.08–2.13]		65%	<0.01
**Mean or median age (y) at inclusion (n = 1772)**				0.44		
<65	1459	(82)	1.57 [1.18–2.11]		77%	<0.01
≥65	313	(18)	1.26 [0.79–2.02]		0%	0.46
**Cancer site**				**<0.01**		
Gastric	458	(15)	0.96 [0.72–1.29]		-	-
Breast	137	(4.5)	2.93 [1.82–4.73]		0%	0.69
Sarcoma	0	(0)	-		-	-
Uterus	0	(0)	-		-	-
Head and neck	862	(29)	2.47 [1.65–3.69]		0%	0.40
Ovarian	0	(0)	-		-	-
Lymphoma	0	(0)	-		-	-
Various	89	(3)				
Cholangiocarcinoma	0	(0)	-		-	-
Melanoma	68	(2)	1.20 [0.40–3.56]		-	-
Leukemia	0	(0)	-		-	-
Colorectal	244	(8)	1.00 [0.80–1.26]		0%	0.56
Anal	0	(0)	-		-	-
Bile ducts	0	(0)	-		-	-
Non-small cell lung	0	(0)	-		-	-
Liver	0	(0)	-		-	-
Myeloma	0	(0)	-		-	-
Thyroids	180	(6)	1.20 [0.89–1.61]		-	-
Pancreatic	281	(9)	1.66 [1.13–2.42]		0%	0.47
Kidney	139	(5)	1.98 [0.99–3.98]		0%	0.71
Esophageal	494	(16.5)	1.17 [0.66–2.08]		86%	<0.01
Urothelial	28	(1)	0.83 [0.21–3.29]		-	-
Prostatic	0	(0)	-		-	-
Small cell lung	0	(0)	-		-	-
**Cancer extension**				**<0.01**		
Various	2216	(74)	1.57 [1.16–2.12]		77%	<0.01
Local	0	(0)	-		-	-
Locally advanced	228	(8)	0.69 [0.47–1.02]		0%	0.62
Metastatic	536	(18)	1.57 [1.18–2.09]		28%	0.22
**Treatment modalities**				0.19		
Not specified	0	(0)	-		-	-
Surgery	0	(0)	-		-	-
Targeted therapy	374	(12.5)	1.63 [1.05–2.54]		30%	0.23
Various	2040	(68.5)	1.22 [0.85–1.74]		79%	<0.01
Immune therapy	68	(2)	1.20 [0.40–3.56]		-	-
Radiotherapy	28	(1)	0.83 [0.21–3.29]		-	-
Chemotherapy	470	(16)	1.98 [1.55–2.54]		32%	0.20
Exclusive supportive care	0	(0)	-		-	-
Intra-arterial infusion for hepatocellular carcinoma	0	(0)	-		-	-
**Definition of sarcopenia**				**<0.01**		
Muscle mass quantity only						
CT scan	2886	(97)	1.53 [1.22–1.93]		70%	<0.01
BIA	94	(3)	0.82 [0.56–1.21]		-	-
DXA	0	(0)	-		-	-
Consensus algorithm-based	0	(0)	-		-	-
**Muscle mass indices (n = 2136)**				-		
AMA (cm^2^)	0	(0)	-		-	-
ASM (kg/m^2^)	0	(0)	-		-	-
TPA (cm^2^/m^2^)	0	(0)	-		-	-
PMI (cm^2^/m^2^)	0	(0)	-		-	-
SMI (cm^2^/m^2^)	2136	(100)	1.49 [1.18–1.90]		69%	<0.01

Bold = grouping data, and significant *p* value at the threshold of 5%; BIA = bioelectrical impedance analysis; DXA = dual energy X-ray absorptiometry; AMA = arm muscle area; ASM = appendicular skeletal muscle mass; SMI = skeletal muscle index; PMI = psoas muscle index; TPA = total psoas area.

**Table 7:** Predictive value of pre-therapeutic sarcopenia on severe treatment-related toxicities and/or dose-limiting toxicities (TOX) among cancer patients.

Study Groups	Patients		Relative Risk [95% CI] for NI	*p* Value for Subgroup Differences	Heterogeneity	
	N	(%)			I^2^	*p*
**Overall**				**<0.01**		
All studies	6246	(100)	1.76 [1.41–2.22]		58%	<0.01
Excluding studies over the 95% CI (funnel plot)	864	(14)	1.15 [0.87–1.52]			
**Quality of study (NOS)**				0.09		
Good	4380	(70)	1.90 [1.45–2.49]		52%	0.01
Fair	1783	(28.5)	1.64 [1.02–2.65]		69%	<0.01
Poor	83	(1.5)	0.83 [0.42–1.65]		-	-
**Year of publication**				0.63		
2008–2012	234	(4)	1.83 [1.03–3.25]		-	-
2013–2017	2128	(34)	1.50 [1.20–1.87]		30%	0.17
2018–2022	3884	(62)	1.87 [1.56–2.23]		72%	<0.01
**N° of patients included**				**<0.01**		
<100	132	(2)	0.76 [0.44–1.32]		0%	0.71
100–199	1059	(17)	1.77 [1.22–2.56]		34%	0.17
200–399	2385	(38)	1.85 [1.23–2.80]		69%	<0.01
≥400	2670	(43)	2.26 [1.66–3.07]		6%	0.36
**World region**				0.33		
Asia	4817	(77)	1.91 [1.42–2.57]		66%	<0.01
Not Asia	1429	(33)	1.53 [1.11–2.12]		36%	0.14
**Mean or median age (y) at inclusion (n = 5047)**				**<0.01**		
<65	2126	(42)	2.60 [1.93–3.52]		41%	0.10
≥65	2921	(58)	1.36 [1.01–1.83]		45%	0.06
**Cancer site**				**<0.01**		
Gastric	3342	(53.5)	2.55 [1.88–3.46]		29%	0.21
Breast	0	(0)	-		-	-
Sarcoma	0	(0)	-		-	-
Uterus	0	(0)	-		-	-
Head and neck	0	(0)	-		-	-
Ovarian	0	(0)	-		-	-
Lymphoma	0	(0)	-		-	-
Various	49	(1)	0.67 [0.27–1.66]		-	-
Cholangiocarcinoma	0	(0)	-		-	-
Melanoma	0	(0)	-		-	-
Leukemia	0	(0)	-		-	-
Colorectal	1033	(16.5)	1.80 [1.31–2.48]		14%	0.33
Anal	0	(0)	-		-	-
Bile-ducts	0	(0)	-		-	-
Non-small cell lung	0	(0)	-		-	-
Liver	0	(0)	-		-	-
Myeloma	0	(0)	-		-	-
Thyroids	0	(0)	-		-	-
Pancreatic	202	(3)	0.69 [0.32–1.49]		-	-
Kidney	0	(0)	0		-	-
Esophageal	1620	(26)	1.49 [1.02–2.18]		61%	0.01
Urothelial	0	(0)	-		-	-
Prostatic	0	(0)	-		-	-
Small cell lung	0	(0)	-		-	-
**Cancer extension**				0.57		
Various	6073	(97)	1.75 [1.38–2.22]		60%	<0.01
Local	173	(3)	2.13 [1.11–4.10]		-	-
Locally-advanced	0	(0)	-		-	-
Metastatic	0	(0)	-		-	-
**Treatment modalities**				0.12		
Not specified	0	(0)	-		-	-
Surgery	6033	(97)	1.84 [1.45–2.32]		58%	<0.01
Targeted therapy	0	(0)	-		-	-
Various	213	(3)	1.05 [0.54–2.06]		19%	0.27
Immune-therapy	0	(0)	-		-	-
Radiotherapy	0	(0)	-		-	-
Chemotherapy	0	(0)	-		-	-
Exclusive supportive care	0	(0)	-		-	-
Intra-arterial infusion for hepatocellular carcinoma	0	(0)	-		-	-
**Definition of sarcopenia**				**0.03**		
-Muscle mass quantity only						
CT-scan	2487	(40)	1.59 [1.28–1.97]		0%	0.45
BIA	423	(7)	1.12 [0.62–2.02]		59%	0.06
DXA	0	(0)	-		-	-
-Consensus algorithm-based	3336	(53)	2.49 [1.75–3.54]		64%	<0.01
**Muscle mass indices (n = 5782)**				0.92		
AMA (cm^2^)	951	(16)	1.86 [1.10–3.16]		-	-
ASM (kg/m^2^)	344	(6)	1.34 [0.44–4.06]		88%	<0.01
TPA (cm^2^/m^2^)	0	(0)	-		-	-
PMI (cm^2^/m^2^)	567	(10)	1.56 [0.73–3.30]		-	-
SMI (cm^2^/m^2^)	3920	(68)	1.85 [1.41–2.43]		53%	0.01

Bold = grouping data, and significant *p* value at the threshold of 5%; BIA = bioelectrical impedance analysis; DXA = Dual Energy X-Ray Absorptiometry; AMA = arm muscle area; ASM = appendicular skeletal muscle mass; SMI = skeletal muscle index; PMI = psoas muscle index; TPA = total psoas area.

## Data Availability

The data presented in this study are available upon request from the corresponding author.

## Appendix A

**Table A1:** Consensus-based algorithm definition of sarcopenia.

Consensus	Year	Screening	Definition				
			Muscle Mass		Muscular Strength		Muscular Performance
**EWGOS 1**	2010	No	↓ DXA, BIA, CT or MRI	**AND**	↓ Hand-grip strength (kg) [M < 30, F < 20]	**OR**	↓ GS < 0.8 m/s or SPPB < 9/12 or TGUG ≥ 20 s
**IWGS**	2011	No	↓ DXA	**AND**	No	**-**	↓ GS < 1 m/s
**AWGS 1**	2014	No	↓ DXA or BIA	**AND**	↓ Hand-grip strength (kg) [M < 26, F < 18]	**AND**	↓ GS < 0.8 m/s
**EWGOS 2**	2019	Yes (SARCF)	↓ DXA, BIA, CT or MRI	**AND**	↓ Hand-grip strength (kg) [M < 27, F < 16] Or 5 Rising from a chair > 15 s	**AND** **(severity)**	↓ GS < 0.8 m/s or SPPB < 9/12 or TGUG ≥ 20 s
**AWGS 2**	2019	Yes (SARCF)	↓ DXA or BIA	**AND**	↓ Hand-grip strength (kg) [M < 28, F < 18] 5 Rising from a chair > 12 s	**AND** **(severity)**	↓ GS < 1 m/s or SPPB < 9/12

EWGOS: European Working Group On Sarcopenia; IWGS: International Working Group on Sarcopenia; AWGS: Asian Working Group on Sarcopenia; DXA: dual-energy X-ray absorptiometry; BIA: bioelectrical impedance analysis; CT: computed tomography; MRI: magnetic resonance imagery. M: male; F: female. GS: gait speed; SPPB: short physical performance battery; TGUG: timed get up and go test. SARCF: strength, assistance with walking, rise from a chair, climb stairs, and falls. ↓: reduced muscle mass; Bold = consensus names, and syndromic combination.

**Table A2:** Multivariate meta-regression of factors significantly influencing the prevalence of pre-therapeutic sarcopenia among cancer patients.

Study Groups	Estimates	Standard Error	*p*
**N° of patients included (n = 65,936)**			
<100	1 (reference)	-	-
100–199	0.02	0.15	0.89
200–300	−0.25	0.16	0.10
≥400	0.51	0.19	**<0.01**
**Definition of sarcopenia**			
Muscle mass only (n = 54,923)	1 (reference)	-	-
Consensus algorithm-based (n = 11,013)	−0.85	0.18	**<0.0001**
**Cut-off values of CT scan-based SMI for women (per 6.0 cm^2^/m^2^ of more)** (n = 14,216)	0.05	0.02	**<0.01**

Bold = grouping data, and significant *p* value at the threshold of 5%.
